# Targeting GSTP1 as Therapeutic Strategy against Lung Adenocarcinoma Stemness and Resistance to Tyrosine Kinase Inhibitors

**DOI:** 10.1002/advs.202205262

**Published:** 2023-01-29

**Authors:** Si‐Qi Wang, Jun‐Jiang Chen, Yuchen Jiang, Zi‐Ning Lei, Ye Chun Ruan, Yihang Pan, Judy Wai Ping Yam, Maria Pik Wong, Zhi‐Jie Xiao

**Affiliations:** ^1^ State Key Laboratory of Stem Cell and Reproductive Biology Institute of Zoology Chinese Academy of Sciences Beijing 100101 China; ^2^ Department of Pathology School of Clinical Medicine The University of Hong Kong Hong Kong SAR 999077 China; ^3^ Institute for Stem Cell and Regenerative Medicine Chinese Academy of Sciences Beijing 100101 China; ^4^ Bejing Institute for Stem Cell and Regenerative Medicine Beijing 100101 China; ^5^ Department of Physiology School of Medicine Jinan University Guangzhou 510000 China; ^6^ Department of Biomedical Engineering Faculty of Engineering The Hong Kong Polytechnic University Hong Kong SAR 999077 China; ^7^ Scientific Research Centre The Seventh Affiliated Hospital Sun Yat‐sen University Shenzhen 518000 China

**Keywords:** calcium/calmodulin‐dependent protein kinase 2 isoform A, cancer stem cells, glutathione S‐transferase pi, lung adenocarcinoma, nuclear factor erythroid 2‐related factor 2

## Abstract

Glutathione S‐transferase *pi* (GSTP1), a phase II detoxification enzyme, is known to be overexpressed and mediates chemotherapeutic resistance in lung cancer. However, whether GSTP1 supports cancer stem cells (CSCs) and the underlying mechanisms in lung adenocarcinoma (LUAD) remain largely unknown. This study unveiled that GSTP1 is upregulated in lung CSCs and supports tumor self‐renewal, metastasis, and resistance to targeted tyrosine kinase inhibitors of LUAD both in vitro and in vivo. Mechanistically, CaMK2A (calcium/calmodulin‐dependent protein kinase 2 isoform A)/NRF2 (nuclear factor erythroid 2‐related factor 2)/GSTP1 is uncovered as a regulatory axis under hypoxia. CaMK2A increased GSTP1 expression through phosphorylating the Sersine558 residue of NRF2 and promoting its nuclear translocation, a novel mechanism for NRF2 activation apart from conventional oxidization‐dependent activation. Upregulation of GSTP1 in turn suppressed reactive oxygen species levels and supported CSC phenotypes. Clinically, GSTP1 analyzed by immunohistochemistry is upregulated in a proportion of LUAD and serves as a prognostic marker for survival. Using patient‐derived organoids from an *ALK*‐translocated LUAD, the therapeutic potential of a specific GSTP1 inhibitor ezatiostat in combination treatment with the ALK inhibitor crizotinib is demonstrated. This study demonstrates GSTP1 to be a promising therapeutic target for long‐term control of LUAD through targeting CSCs.

## Graphical abstract

This study unveils the role of  calcium/calmodulin‐‐depdendent protein kinase 2 isoform A (CaMK2A)/nuclear factor erythroid 2‐related factor (NRF2)/glutathione S‐transferase pi (GSTP1) axis under hypoxia in lung cancer stem cell maintenance. GSTP1 inhibitor Ezastiostat exhibits the potential as a combined treatment with tyrosine kinase inhibitor (TKI) to reverse TKI resistance, illustrating GSTP1 as promising therapeutic target for lung adenocarcinoma.

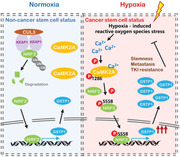

## 1 Introduction

Cancer stem cells (CSCs) are a subset of cancer cells with enhanced capacities for self‐perpetuation and differentiation into different cell lineages, believed to initiate tumor growth and promote cancer progression.^[^ ^1^ ^]^ The conversion from non‐CSCs to CSCs is enabled by phenotypic plasticity in response to stimuli from tumor microenvironment such as oxidative stress.^[^ ^2^ ^]^ Constitutive regulation of such stresses is crucial for maintenance of metabolic homeostasis and CSC properties.^[^ ^3^ ^]^ Studies have demonstrated CSC have a significantly lower level of reactive oxygen species (ROS) than non‐CSCs, which in turn enhances self‐renewability more than cells with higher ROS levels.^[^ ^4^ ^]^ The role of hypoxia and ROS in CSCs enrichment and sustenance has been reported in multiple cancers including glioblastoma, colorectal, and lung cancer.^[^ ^5^ ^]^ The unveiling of new regulatory mechanisms of hypoxia and redox equilibrium could drive advances in lung cancer treatment.

Glutathione S‐transferases (GSTs), a super‐enzyme family including at least eight classes of isoenzymes, mediate strong cytoprotective capacities by eliminating toxic substances such as xenobiotics, carcinogens, and electrophiles through catalytic conjugation to glutathione.^[^ ^6^ ^]^ Intracellular glutathione metabolism has both beneficial and pathogenic impacts on several types of cancers. Appropriate level of glutathione enhance cell survival, whereas excessive glutathione facilitates tumor metastasis, indicating the potential important role of GSTs in modulating carcinogenesis and tumor progression.^[^ ^7^ ^]^ Glutathione S‐transferase *pi* (GSTP1) is the only isoenzyme of the *pi* class that has been reported to be over‐expressed in several cancer types including lung cancer.^[^ ^8^ ^]^ Serving as a phase II detoxification enzyme, its functions on detoxifying chemotherapeutic agents have been widely reported, but other functions of GSTP1 on cancer cells regulation are less reported.^[^ ^9^ ^]^ In breast cancer, GSTP1 has been reported to facilitate tumorigenesis by regulating glycolytic metabolism.^[^ ^10^ ^]^ In colorectal cancer, simultaneous inhibition of GSTP1 and thioredoxin reductase reduced the proportion of CD44V8‐10^+^ CSC‐like cells,^[^ ^11^ ^]^ indicating the potential effects of GSTP1 on regulating CSC properties. Overexpression of GSTP1 in lung CSCs and its expected function in cisplatin resistance has been acknowledged.^[^ ^12^ ^]^ Since cisplatin is a well‐known substrate of GSTP1, induction of cisplatin‐resistance provides very limited support to demonstrate the effects of GSTP1 on lung CSC regulation. Whether and how GSTP1 regulates cardinal lung CSC properties, including stemness, aggressiveness, and resistance to non‐substrate anti‐cancer treatment are still unknown.

In this study, using patient‐derived lung adenocarcinoma (LUAD) organoids and genetically modulated cancer cell lines, we provide data to uncover an unrecognized hypoxia/calcium/calmodulin‐dependent protein kinase 2 isoform A (CaMK2A)/nuclear factor erythroid 2‐related factor 2 (NRF2)/GSTP1 axis in lung cancer and demonstrate its role in CSC regulation. Under hypoxia, GSTP1 expression is enhanced through transcriptional regulation by NRF2, which is activated by the CaMK2A via Serine558 (S558)) phosphorylation. Functionally, activation of the CaMK2A/NRF2/GSTP1 axis restores redox homeostasis through negative feedback. GSTP1 regulates multifaceted CSC phenotypes in lung cancer.

Lung cancer carries the highest incidence and mortality of all malignancies worldwide. Non‐small cell lung cancer is found in about 85% of all lung cancers, with LUAD being the most common subtype.^[^ ^13^ ^]^ The overall 5 years survival is less than 25% due to late presentation and drug resistance.^[^ ^1b^ ^]^ Tyrosine kinase inhibitors (TKIs) are first line treatment for LUAD patients harboring *EGFR* mutations or *ALK* rearrangements.^[^ ^14^ ^]^ However, drug resistance is inevitable event for almost all of these patients. Targeting CSCs in combination with TKIs may provide extra armamentarium to improve cancer survival. By using *ALK*‐translocated LUAD and induced TKI‐resistant LUAD organoids in the current study, we demonstrate the promising role of ezatiostat, an FDA‐approved GSTP1 inhibitor, in single agent treatment of LUAD or in combination with ALK targeted therapy, indicating that GSTP1 is an effective therapeutic target of LUAD.

## 2 Results

### 2.1 GSTP1 was Upregulated in CSC and Supported Stemness of LUAD

**Figure 1:**
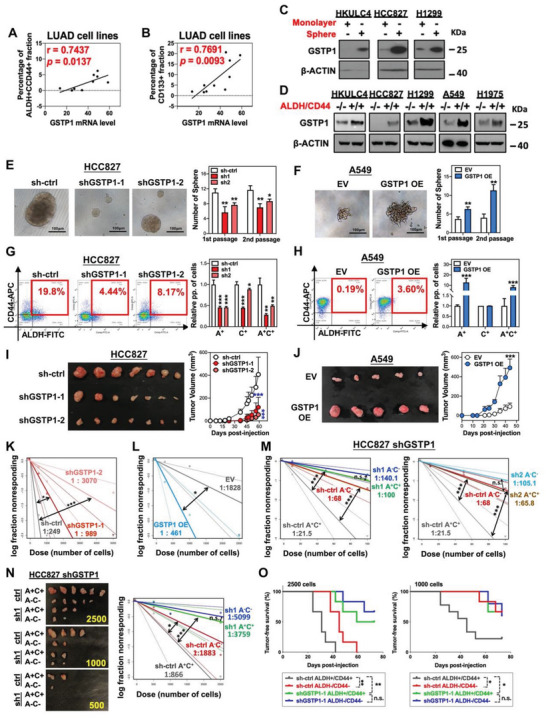
GSTP1 was upregulated in CSC and supported stemness and metastasis properties in LUAD. A,B) Correlation of GSTP1 mRNA level with ALDH^+^/CD44^+^ (A) and CD133^+^ (B) CSC populations, respectively, in LUAD cell lines analyzed by Pearson correlation test. C) Western blot analysis of GSTP1 expression in tumorspheres and corresponding monolayers derived from HKULC4, HCC827, and H1299 cells. D) Western blot analysis of GSTP1 expression in ALDH^+^/CD44^+^ CSC and ALDH^−^/CD44^−^ non‐CSC fractions sorted from HKULC4, HCC827, H1299, A549, and H1975 cells. E,F) Tumorspheres serially passaged for two generations using HCC827 cells with GSTP1‐KD (E), and A549 cells with GSTP1‐OE (F), showing representative images of tumorspheres (left) and histograms of sphere numbers (right). G,H) Proportions of ALDH^+^/CD44^+^ subsets in HCC827 cells with or without GSTP1‐KD (G), and A549 cells with or without GSTP1‐OE (H). I,J) In vivo tumorigenicity in SCID mice evaluated by subcutaneous injection of HCC827 cells with or without GSTP1‐KD (I), and A549 cells with or without GSTP1‐OE (J), showing representative images of xenografts (left) and the corresponding tumor growth curves (right). K,L) In vivo limiting dilution assays for frequency of CSCs in HCC827 cells with GSTP1‐KD (K) and A549 cells with GSTP1‐OE (L). CSC frequencies and *p* values were calculated using the ELDA online tool (https://bioinf.wehi.edu.au/software/elda/). M) In vitro limiting dilution assay for frequency of CSCs in HCC827 ALDH^+^/CD44^+^ CSC and ALDH^−^/CD44^−^ non‐CSC fractions with or without GSTP1‐KD, respectively. N) In vivo limiting dilution assay for frequency of CSCs in HCC827 ALDH^+^/CD44^+^‐CSC and ALDH^−^/CD44^−^ non‐CSC fractions with or without GSTP1‐KD, respectively. Representative images of xenografts (left) and the corresponding CSC frequencies (right) are shown. O) Kaplan–Meier curve and log‐rank test showed tumor‐free survival of SCID mice injected with 2500 and 1000 cells, respectively. Data are presented as mean ± SD of triplicate measurements. * *p* < 0.05, ** *p* < 0.001, *** *p* < 0.005 versus respective control by Student's *t*‐test.

Both the protein and mRNA levels of GSTP1 in a LUAD cell line panel normalized to those of the immortalized bronchial epithelial cell line BEAS‐2B showed elevation according to western blot and qPCR, respectively (Figure S1A,B, Supporting Information), indicating its potential oncogenic roles in LUAD. ALDH/CD44 co‐expression and CD133 are well established CSC markers for lung cancer.^[^ ^15^ ^]^ The correlation of GSTP1 mRNA level and LUAD CSC markers was analyzed in the LUAD cell line panel. Results showed that GSTP1 mRNA level was significantly and positively correlated with ALDH^+^/CD44^+^ CSC fractions and CD133^+^ CSC fractions with correlation coefficients of 0.7437 and 0.7691, respectively (**Figure** **1**A,B; Figure S1C,D, Supporting Information). Moreover, GSTP1 was expressed at higher levels in tumorspheres, a CSC surrogate, compared with that in the non‐CSC surrogate of matched monolayered cells (Figure 1C). By using ALDH and CD44 co‐expression as CSC markers, western blot showed the ALDH^+^/CD44^+^ CSC population displayed higher GSTP1 levels compared to ALDH^−^/CD44^−^ non‐CSCs of several LUAD cell lines (Figure 1D), suggesting the supportive role of GSTP1 in CSC regulation. Next, functional studies were conducted by knocking down GSTP1 (GSTP1‐KD) in HCC827 and H1975 cells using two distinct short‐hairpin RNAs (sh1 and sh2), or by GSTP1 stable overexpression (GSTP1‐OE) in A549 cells (Figure S1E, Supporting Information). GSTP1‐KD significantly attenuated tumorsphere formation and the ALDH^+^/CD44^+^ fraction without suppressing cellular proliferation rate, and increasing apoptosis rate and senescence in HCC827 and H1975 cells whereas ectopic expression led to the opposite effects (Figure 1E–H; Figure S1F–L, Supporting Information). In addition, GSTP1 manipulation exerted differential effects on CD44 variants. Only the expression of *CD44V8‐10* variant was significantly and consistently suppressed in HCC827 GSTP1‐KD cells and elevated in A549 GSTP1‐OE cells, while other CD44 variants showed inconsistent changes (Figure S1M–P, Supporting Information).

Furthermore, the in vivo effects of GSTP1 were studied using subcutaneous xenograft models. GSTP1‐KD significantly reduced tumor growth rate and volume, whereas GSTP1‐OE promoted tumor formation (Figure 1I,J; Figure S2A, Supporting Information). In vivo limiting dilution assays showed that, compared with the matched control group, silencing of GSTP1 by sh1 and sh2 leads to a 3‐fold and 19‐fold decrease in CSC frequency, respectively, whereas upregulation of GSTP1 caused a 3‐fold increase in CSC frequency (Figure 1K,L; Figure S2B,C, Supporting Information). To further evaluate the specific effects of GSTP1 on LUAD stemness regulation, in vitro and in vivo limiting dilution assays were conducted in ALDH^+^/CD44^+^ and ALDH^−^/CD44^−^ fractions isolated from HCC827 with or without GSTP1‐KD, respectively. In vitro limiting dilution assay showed that, compared with the sh‐ctrl‐ALDH^+^/CD44^+^ fraction, CSC frequency was significantly reduced in both sh‐ctrl‐ALDH^−^/CD44^−^ and shGSTP1‐ALDH^+^/CD44^+^ fractions. Notably, the CSC frequencies were not significantly different in the shGSTP1‐ALDH^+^/CD44^+^ and sh‐GSTP1‐ALDH^−^/CD44^−^ fractions, indicating the prominent effects of GSTP1 on maintaining the CSC pool in LUAD (Figure 1M). Consistently, in vivo limiting dilution assay showed, compared with the sh‐ctrl‐ALDH^+^/CD44^+^ fraction, the sh‐ctrl‐ALDH^−^/CD44^−^ fraction and shGSTP1‐ALDH^+^/CD44^+^ fractions showed significantly reduced CSC frequency, tumor incidence, and longer tumor‐free survival, respectively. No significant differences were found in the CSC frequencies and tumor‐free survival between shGSTP1‐ALDH^+^/CD44^+^ fraction and shGSTP1‐ALDH^−^/CD44^−^ fraction (Figure 1N,O; Figure S2D, Supporting Information). Overall, the results indicated that GSTP1 played a supportive role for stemness in LUAD.

### 2.2 GSTP1 Facilitated Tumor Metastasis in LUAD

**Figure 2:**
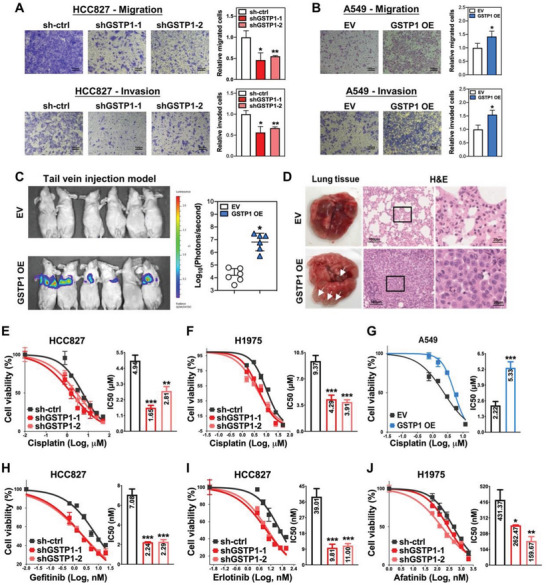
GSTP1 enhanced metastasis and LUAD resistance to anti‐cancer drugs. A,B) Transwell migration (upper panel) and invasion (lower panel) assays performed in HCC827 (A) and A549 (B) cells with or without GSTP1 manipulation. Histograms showed the relative migrated and invaded proportions of the seeded cells compared to the control group. C) In vivo tail vein injection model for the evaluation of tumor metastasis with or without GSTP1‐OE. Luciferase labeled A549 cells (2 × 10^6) were injected into nude mice through tail veins, and bioluminescence imaging was performed after 10 weeks. In vivo bioluminescence images of mice injected with GSTP1‐OE or empty vector (EV) control cells (left). Scatter plot for the quantitative comparison of bioluminescence signals in respective groups (right). D) Representative images of lung harvested at the 10th week after injection of A549 cells with GSTP1‐OE or EV control, featuring gross images of harvested lung with white arrows indicating tumor nodules (left), H&E stained histology of random areas of control lung (EV) and tumor nodules of GSTP1‐OE cells (middle), and high power view of the marked areas in the middle panel (right), respectively. E,F) Cell viability by MTT assay of scramble control and GSTP1‐KD cells treated with cisplatin, using HCC827 (E), and H1975 (F) cells. G) Cell viability assay for cisplatin treatment by MTT comparing A549 cells with GSTP1‐OE and EV control. H,I) Cell viability assay by MTT comparing effects of HCC827 control and GSTP1‐KD cells treated with gefitinib (H) or erlotinib (I). J) Effects of GSTP1‐KD on afatinib sensitivity of H1975 cells by MTT assay. Data represented mean ± SD of triplicate measurements. * *p* < 0.05, ** *p* < 0.001, *** *p* < 0.005 versus respective control by Student's *t*‐test.

In vitro cell mobility studies showed GSTP1‐KD significantly inhibited migration and invasion of both HCC827 and H1975 cells (**Figure** **2**A; Figure S2E, Supporting Information), while ectopic GSTP1‐OE in A549 cells resulted in opposite effects (Figure 2B). In vivo analysis using nude mice and tail vein injection of luciferase‐labeled A549 GSTP1‐OE or control (EV) cells showed, by the 10th week post‐injection, 5/6 mice in the GSTP1‐OE group developed bioluminescent lung nodules which were histologically confirmed to be adenocarcinomas (Figure 2C,D), while 0/6 control mice developed tumors, respectively. Moreover, western blot studies of A549 and H1299 cells showed that GSTP1‐OE inhibited the expression of the epithelial cell adhesion marker E‐cadherin, and increased that of the mesenchymal marker VIMENTIN compared to control (Figure S2F, Supporting Information), indicating GSTP1 facilitated cancer metastasis through regulating epithelial–mesenchymal transition (EMT).

### 2.3 GSTP1 Enhanced LUAD Resistance to Anti‐Cancer Drugs

The most resilient CSCs are known to contribute to drug resistance through adaptation to the selective force of anti‐cancer treatment, regardless of variations in genetic background of cancers.^[^ ^16^ ^]^ Therefore, we investigated the role of GSTP1 in drug resistance. In line with the reported action of GSTP1 in drug detoxification,^[^ ^17^ ^]^ GSTP1‐KD or ‐OE led to diminution or augmentation of resistance to cisplatin chemotherapy, respectively (Figure 2E–G). Interestingly, analysis of the effect of EGFR tyrosine kinase inhibitors (TKIs) on HCC827 (*EGFR* exon 19 deletion) cells showed that GSTP1‐KD led to higher sensitivity to gefitinib and erlotinib (Figure 2H,I). In H1975 cells that harbors concurrent *EGFR L858R* and *T790M*, GSTP1 silencing also enhanced sensitivity to the second generation TKI afatinib compared with control cells (Figure 2J). Since TKIs are not the known substrates of GSTP1, these data supported the notion that GSTP1 might mediate TKI resistance through enhancing CSC resilience of LUAD.

### 2.4 GSTP1 Upregulation was Mediated by CaMK2A/NRF2 Axis

**Figure 3:**
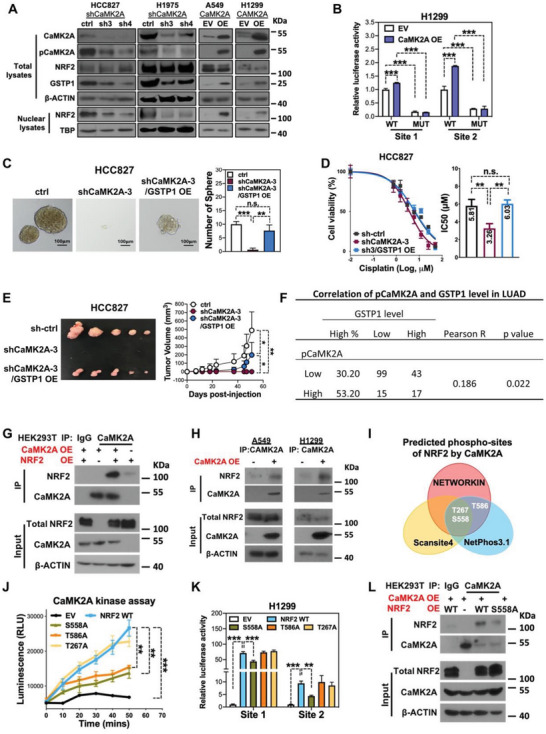
GSTP1 upregulation was mediated by CaMK2A/NRF2 S558 axis. A) Western blot analyses of protein levels in LUAD cells with or without CaMK2A manipulation, including CaMK2A, pCaMK2A T286, NRF2, GSTP1, and nuclear NRF2. B) Relative luciferase activities of GSTP1 reporters with wild type (WT) or mutant NRF2‐binding sites in H1299 cells with or without CaMK2A‐OE. C–E) Effects of GSTP1‐OE in HCC827 cells with or without CaMK2A‐KD, with respect to tumorspheres (C), cell viability under cisplatin treatment (D), and in vivo tumorigenesis (E). F) Correlation of pCaMK2A and GSTP1 level in 178 clinical LUAD cases analyzed by Pearson correlation, and the significance was tested by the Chi‐square test. G) Co‐immunoprecipitation (Co‐IP) of NRF2 in HEK293T cells with CaMK2A‐OE and/or NRF2‐OE, using CaMK2A as bait. H) Co‐IP of NRF2 in A549 and H1299 cells with or without stable CaMK2A‐OE using CaMK2A as bait. I) Venn diagram of prediction of NRF2 phosphorylation sites by CaMK2A using on‐line databases of NETWORKIN, NetPhos3.1 and Scansite4 showed that NRF2 T267, S558, and T586 were the candidates. J) Time‐dependent CaMK2A kinase assay with NRF2 WT or mutant protein precipitated from total cell lysate of HEK293T cells with exogenously overexpressed empty vector, NRF2 WT, NRF2 T267A, NRF2 S558A, or NRF2 T586A, respectively. K) Relative luciferase activities of *GSTP1* reporter with wild type NRF2‐binding sites in H1299 cells exogenously forced to overexpress empty vector, NRF2 WT, NRF2 T267A, NRF2 S558A, or NRF2 T586A, respectively. L) Co‐IP of NRF2 in HEK293T cells overexpressing CaMK2A concurrent with either NRF2 WT or NRF2 S558A using CaMK2A as bait. Data represented mean ± SD of triplicate measurements. * *p* < 0.05, ** *p* < 0.001, *** *p* < 0.005 versus respective control by Student's *t*‐test.

We previously observed that CaMK2A promoted CSC in LUAD while NRF2 activation is known to trans‐regulate GSTP1,^[^ ^18^ ^]^ suggesting CaMK2A might mediate GSTP1 expression through regulating NRF2 transcriptional activity. To support our hypothesis, the expression levels of GSTP1 and nuclear NRF2 in CaMK2A manipulated cell lines were first evaluated by western blot. Results showed that forced expression of CaMK2A in A549 and H1299 cells led to an increase in GSTP1 and nuclear NRF2 levels without affecting total NRF2 level, whereas CaMK2A‐KD in both HCC827 and H1975 resulted in downregulation of both nuclear NRF2 and GSTP1 levels (**Figure** **3**A), suggesting CaMK2A might upregulate GSTP1 through promoting NRF2 nuclear translocation. To further investigate this possibility, the expression of CaMK2A/NRF2/GSTP1 axis was further compared in CSC and non‐CSC fractions. Western blot showed that pCaMK2A level was upregulated in both tumorspheres and ALDH^+^/CD44^+^‐CSC compared to monolayer cells and ALDH^−^/CD44^−^ non‐CSC fraction, respectively, in several LUAD cell lines (Figure S3A,B, Supporting Information). Furthermore, immunofluorescence staining showed that more NRF2s were translocated into the nuclei in ALDH^+^/CD44^+^ CSC fraction than in ALDH^−^/CD44^−^ non‐CSC fraction, indicating that CaMK2A/NRF2/GSTP1 axis was activated in LUAD CSC (Figure S3C, Supporting Information). The specificity of NRF2 antibody was verified in HCC827 cells with NRF2‐KD (Figure S4A, Supporting Information).

To further demonstrate the CaMK2A/NRF2/GSTP1 regulatory axis, wild type or mutated reporters spanning two distinct NRF2‐binding sites on *GSTP1* (−71 and +2129 bp from the transcription start site, respectively) were designed (Figure S4B, Supporting Information), and their specificity were verified by dual luciferase assay in both H1299 and A549 cells with or without NRF2 ectopic expression (Figure S4C,D, Supporting Information). Furthermore, the involvement of NRF2 in CaMK2A/GSTP1 axis was investigated by measuring the luciferase activities of these reporters in H1299 and A549 cells with or without CaMK2A‐OE, respectively. Luciferase activities from GSTP1 reporters with wild type NRF2 binding site were significantly higher in H1299 and A549 CaMK2A‐OE cells compared to control cells, respectively, but annihilated in cells with NRF2 binding site mutated reporters regardless of CaMK2A levels (Figure 3B; Figure S4E, Supporting Information). Together, the results suggested GSTP1 can be trans‐regulated by CaMK2A/NRF2 signaling.

To explore the functional roles of CaMK2A on GSTP1 expression, we ectopically expressed GSTP1 in HCC827 cells with CaMK2A‐KD. Restoration of GSTP1 in CaMK2A‐KD cells significantly rescued self‐renewability, cisplatin‐resistance, and in vivo tumorigenicity repressed by CaMK2A silencing (Figure 3C–E; Figure S4F, Supporting Information). Reciprocally, we ablated GSTP1 expression in A549 cells with CaMK2A‐OE. A549 cells with CaMK2A‐OE exhibited enhanced capacities of tumorspheres formation and cisplatin‐resistance, whereas knockdown of GSTP1 in A549 CaMK2A‐OE cells significantly suppressed tumorspheres and restored cisplatin sensitivity (Figure S4G–I, Supporting Information), indicating GSTP1 is involved in CaMK2A‐sustained CSC properties and is regulated by CaMK2A/NRF2 axis. The correlation of CaMK2A and GSTP1 was further evaluated in our clinical cohort, and data showed that CaMK2A levels were significantly and positively related to GSTP1 expression in 178 clinical LUAD cases (Figure 3F). We also studied the correlation between CaMK2A/NRF2 expression and GSTP1 expression in a lung cancer cell line panel. The expression of pCaMK2A was correlated with GSTP1 expression (Figures S1A and S4J, Supporting Information), supporting the regulatory role of CaMK2A on GSTP1. However, both the total and nuclear NRF2 levels in these cell lines did not correlate with pCaMK2A/GSTP1 level (Figures S1A and S4J, Supporting Information), which indicated factors other than CaMK2A might regulate the expression and activation of NRF2.

As NRF2 is a key stress‐sensitive transcription factor involved in regulating the expression of a series of anti‐oxidant and detoxification genes including *HOMX1*, *GCLC*, *GLCM*, and *NQO1*, whether CaMK2A specifically regulates the transcription of GSTP1 was studied. The mRNA levels of *GSTP1*, *GCLC*, *HOMX1*, *NQO1*, and *TRXR1* in CaMK2A manipulated cells detected by qPCR assay showed only *GSTP1* was significantly and consistently downregulated in cells with CaMK2A‐KD (Figure S4K, Supporting Information), suggesting the specific regulatory role of CaMK2A/NRF2 axis in GSTP1 expression.

### 2.5 CaMK2A Enhanced NRF2 Transcription Activity via Direct Phosphorylating NRF2 at S558 Residue

We further explored the mechanism of NRF2 activation by CaMK2A. Since CaMK2A has not been reported to activate NRF2, we hypothesized that NRF2 might be a substrate of CaMK2A. First, co‐immunoprecipitation (Co‐IP) experiments using CaMK2A or isotype‐matched control as the bait were employed to explore this possibility. In HEK293T cells with or without CaMK2A and/or NRF2 overexpression, higher levels of NRF2 were precipitated in cells with CaMK2A and NRF2 co‐overexpression compared to those overexpressing NRF2 only or control cells with empty vectors (Figure 3G). Additionally, in A549 and H1299 cells, CaMK2A‐OE resulted in an increased NRF2 precipitant by anti‐CaMK2A compared with empty‐vector control (Figure 3H). Together, the findings supported a physical interaction between NRF2 and CaMK2A. Next, to determine potential NRF2 phosphorylation residues by CaMK2A, prediction was carried out by using three on‐line phospho‐sites prediction databases. NETWORKIN^[^ ^19^ ^]^ (http://networkin.science/) predicted 7 potential phosphorylated residues of NRF2 by CaMK2A with score over 0.8, NetPhos3.1^[^ ^20^ ^]^ (https://services.healthtech.dtu.dk/service.php?NetPhos‐3.1) listed 21 potential residues with 0.45 as cut off, and Scansite4^[^ ^21^ ^]^ showed 2 potential residues (https://scansite4.mit.edu/), with T267, S558, and T586 being the common candidates (Figure 3I; Figure S5A, Supporting Information). To corroborate this prediction, phospho‐deficient NRF2 was generated by substituting serine with alanine at the 267th, 558th, and 586th residues, respectively (NRF2 T267A, S558A, and T586A). In vitro CaMK2A kinase assay was performed to examine whether CaMK2A phosphorylated NRF2 at predicted residues. Kinase reaction was conducted by incubating recombinant CaMK2A with purified WT or mutant NRF2 protein for indicated times. Compared to the incubation with precipitant from empty vector control lysate, kinase activity of CaMK2A increased time dependently in the presence of precipitated WT NRF2 protein. However, this phosphorylation reaction was significantly inhibited in a time‐dependent manner when phospho‐deficient mutation was introduced at NRF2 S558 and NRF2 T586 residues, while no inhibitory effect was observed for NRF2 T267A mutant (Figure 3J). The functional role of phosphorylating NRF2 T267, S558, and T586 on *GSTP1* trans‐regulation was further demonstrated using dual luciferase assay in H1299 and A549 cells by overexpression. Overexpression of WT NRF2 led to a significant increase in transcriptional activity of GSTP1 reporters, phospho‐deficient mutation at S558 (S558A) significantly suppressed these transcription promoting effects, while T267A and T586A did not significantly alter the luciferase activities relative to WT NRF2 (Figure 3K; Figure S5B, Supporting Information). Furthermore, Co‐IP using anti‐CaMK2A showed reduced NRF2 precipitation in cells overexpressing NRF2 S558A compared to cells overexpressing WT NRF2 (Figure 3L). To further study whether NRF2 S558 phosphorylated by CaMK2A promoted NRF2 nuclear translocation, we generated phosphomimic mutant NRF2 by changing serine into aspartic acid at NRF2 558 residue (NRF2‐S558D) and performed immunofluorescence staining assay on H1299 cells overexpressing WT‐NRF2, NRF2‐S558A, and NRF2‐S558D, respectively. Results showed that NRF2‐S558D exhibited strong nuclear staining, whereas abundant NRF2‐S558A signals were localized in the cytoplasm with reduced nuclear staining compared to the signal of WT‐NRF2 (Figure S5C, Supporting Information). Moreover, GSTP1 was significantly upregulated in H1299 with WT‐NRF2 expression which was further augmented by forced overexpression of NRF2‐S558D but reduced by NRF2‐S558A (Figure S5D, Supporting Information). To further address whether NRF2‐S558D could rescue GSTP1 expression in CaMK2A‐KD cells, we stably overexpressed WT‐NRF2, NRF2‐S558A, and NRF2‐S558D in HCC827 CaMK2A‐KD cells, respectively. Western blot showed that overexpression of WT‐NRF2 increased GSTP1 level in CaMK2A‐KD cells. Overexpression of NRF2‐S558A failed to rescue GSTP1 level, while NRF2‐S558D overexpression further enhanced the expression of GSTP1 compared to WT‐NRF2 (Figure S5E, Supporting Information). Together, these results validated that CaMK2A regulated GSTP1 expression by directly phosphorylating NRF2 at S558.

Since NRF2 is canonically activated by its quencher KEAP1, we also explored whether CaMK2A activated NRF2 in a KEAP1‐dependent manner. KEAP‐KD in both H1299 control cells and CaMK2A‐OE cells led to an increase in the total and nuclear NRF2 to a comparable level. While CaMK2A‐OE increased the expression of GSTP1, KEAP‐KD did not alter GSTP1 expression in both the control and CaMK2A‐OE H1299 cells, indicating that CaMK2A mediated KEAP1 independent regulation in GSTP1 expression (Figure S5F, Supporting Information). To further study the effects of KEAP1 on CaMK2A/NRF2/GSTP1 axis, luciferase reporter assay was carried out. Silencing of KEAP1 in H1299 cells with or without CaKM2A‐OE significantly augmented the transcription activity of GSTP1 reporter site 1, but significantly suppressed that of GSTP1 reporter site 2 (Figure S5G, Supporting Information), suggesting differential regulatory roles of KEAP1 on various regulatory regions of GSTP1. Thus, the data indicated CaMK2A regulated the NRF2/GSTP1 axis in a KEAP1‐independent manner. Together, our results indicated CaMK2A could upregulate GSTP1 through phosphorylating NRF2 at S558, forming a functional CaMK2A/NRF2/GSTP1 axis that supported CSC phenotypes in LUAD.

### 2.6 Hypoxia Activated the CaMK2A/NRF2/GSTP1 Axis

**Figure 4:**
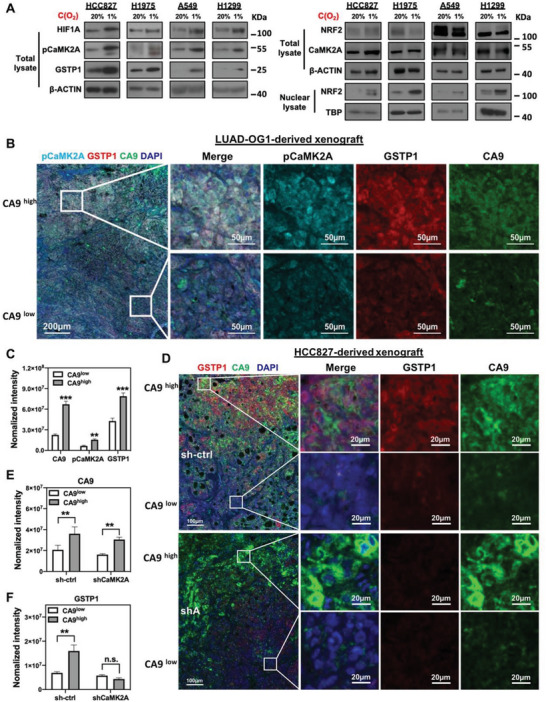
Hypoxia activated the CaMK2A/NRF2/GSTP1 axis. A) Western blot analysis of HIF1A, NRF2, CaMK2A, pCaMK2A T286, GSTP1, and nuclear NRF2 expressions in LUAD cell lines incubated under normoxic (20% O_2_) or hypoxic (1% O_2_) condition. B) Representative images of multiplex IHC staining for pCaMK2A, GSTP1, and CA9 in LUAD‐OG1‐derived xenografts, comparing relatively hypoxic and non‐hypoxic regions at low power (left‐most photomicrograph). High power views (other panels) of marked areas, comprising relatively hypoxic regions with CA9^high^ staining (upper), and relatively non‐hypoxic regions with CA9^low^ staining (lower). C) Histogram comparison of normalized fluorescence intensities of CA9, pCaMK2A, and GSTP1 signals of multiplex IHC staining from CA9 high and CA9 low regions of LUAD‐OG1‐derived xenografts. D) Representative images of multiplex IHC staining of xenografts from HCC827 control (sh‐ctrl, upper panel) or CaMK2A‐KD cells (sh3, lower panel), comparing relatively hypoxic (CA9^high^) and non‐hypoxic (CA9^low^) regions. E,F) Normalized fluorescence intensities of CA9 and GSTP1 signal of multiplex IHC staining performed in FFPE xenograft tissue derived from HCC827 cells with or without CaMK2A‐KD. Color scheme: pCaMK2A (Cyan; Opal670), GSTP1 (Red; Opal570), CA9 (Green; Opal520), and DAPI (Blue; DAPI). ** *p* < 0.001, *** *p* < 0.005 versus corresponding control by Student's *t*‐test.

Incubation of several LUAD cell lines in a hypoxic tumor microenvironment can induce CSC markers and phenotypes in lung cancer.^[^ ^5^, ^22^ ^]^ We thus postulated hypoxia could induce CSC phenotypes through the CaMK2A/NRF2/GSTP1 axis. Hypoxia induced by both 1% O_2_ and CoCl_2_ treatment significantly upregulated mRNA levels of the CSC markers *ALDH1* and *CD44V8‐10* (Figure S6A–D, Supporting Information), supporting the notion that hypoxia promoted the enrichment of CSC fractions. Effects of hypoxia on stimulating CaMK2A/NRF2/GSTP1 axis were further investigated. Successful induction of hypoxia by 1% O_2_ and CoCl_2_ was verified by an increased expression of HIF1A. It was shown that CaMK2A was activated with increased pCaMK2A T286 (pCaMK2A) and GSTP1 levels, concurrent with enhanced NRF2 nuclear accumulation in multiple cell lines (HCC827, H1975, A549, and H1299) (**Figure** **4**A). Similar results were obtained in cells treated with the hypoxia mimicking agent CoCl_2_ (Figure S6E, Supporting Information).

The in vivo involvement of hypoxia in CaMK2A and GSTP1 stimulation was demonstrated using multiplex fluorescent immunohistochemistry (IHC) staining. In xenografts derived from patient‐derived organoids of LUAD (LUAD‐OG1), concurrent increase in activated pCaMK2A and GSTP1 staining was observed in hypoxic regions identified by high carbonic anhydrase 9 (CA9) levels^[^ ^23^ ^]^ (Figure 4B,C). Moreover, in xenografts derived from HCC827 control cells, higher level of GSTP1 was observed in CA9^high^ regions compared to CA9^low^ regions. In those derived from CaMK2A‐KD cells, no significant differences in GSTP1 expression was observed between such regions (Figure 4D–F). Consistently, in HCC827 cells, hypoxia induced GSTP1 upregulation in control cells but knockdown of CaMK2A prevented this (Figure S6F, Supporting Information). Together, these findings supported hypoxia induced CSC marker expression and activation of CaMK2A/NRF2/GSTP1 axis.

### 2.7 CaMK2A/GSTP1 Axis Enhanced Cancer Cell Stemness and Drug Resistance through ROS Suppression

**Figure 5:**
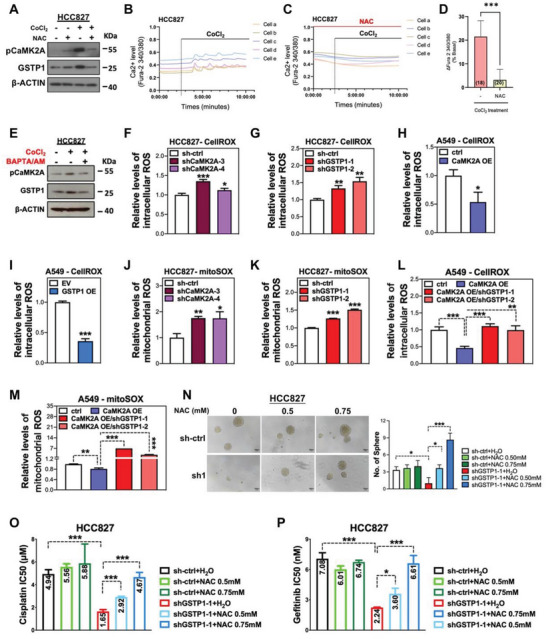
CaMK2A/GSTP1 axis enhanced LUAD stemness and drug resistance through ROS suppression. A) Western blot analysis of pCaMK2A and GSTP1 expressions in cells with or without 100 µm CoCl_2_ and/or 5 mm NAC treatment. B,C) Effect of NAC (1 mm) on CoCl_2_‐induced intracellular Ca^2+^ and oscillations measured by Fura‐2 in HCC827 cells. D) Number of measurements and average intensity of oscillation was shown in each column. E) Western blot analysis of pCaMK2A and GSTP1 expressions in cells with or without 100 µm CoCl_2_ and/or 1 µm of the calcium chelator BAPTA/AM. F–I) Relative intracellular ROS levels of HCC827 cells with or without CaMK2A‐KD (F) or GSTP1‐KD (G), and A549 cells with or without CaMK2A‐OE (H) or GSTP1‐OE (I) detected by CellRox dye and flow cytometry. J–M) Relative mitochondrial ROS levels of HCC827 cells with or without CaMK2A‐KD (J) and GSTP1‐KD (K) detected by mitoSOX dye and flow cytometry. Relative intracellular ROS levels detected by CellROX dye (L) and mitochondrial ROS levels detected by mitoSOX dye (M) in A549 cells with or without CaMK2A and GSTP1 manipulation. N) The effects of the ROS scavenger NAC on tumorspheres formation of HCC827 cells with or without GSTP1‐KD. The left panel showed representative images of tumorspheres (left) and histograms of sphere numbers (right). O,P) The effects of NAC, with or without GSTP1‐KD, on cell viability by MTT assay of HCC827 cells treated with cisplatin (O), or gefitinib (P). Data represented mean ± SD of triplicate measurements. * *p* < 0.05, ** *p* < 0.001, *** *p* < 0.005 versus respective control by Student's *t*‐test.

Hypoxia is one of the key features of the CSC niche which endows CSC with aggressive properties and facilitates the activation of CSC adaptive response against oxidative stress.^[^ ^24^ ^]^ We hypothesize that hypoxia‐induced oxidative stress enriches CSC phenotypes through activating the CaMK2A/GSTP1 axis, which in turn acts as a defensive mechanism against the increased ROS level so that the oxidative stress level of CSCs could be maintained at an optimal level. Consistent with our hypothesis, our results showed that hypoxia mimicked by CoCl_2_ treatment increased expression of pCaMK2A and GSTP1. Hypoxia‐induced CaMK2A activation and GSTP1 elevation were prevented by addition of the ROS scavenger *N*‐acetyl cysteine (NAC) (**Figure** **5**A), suggesting hypoxia induced CaMK2A/GSTP1 activation through increase in oxidative stress. Consistently, the mRNA levels of CSC markers *CD44V8‐10* and *ALDH1* were increased under hypoxia, but suppressed by treating with NAC (Figure S7H,I, Supporting Information), indicating hypoxia‐induced ROS stimulated the CSC plasticity through activating the CaMK2A/GSTP1 axis. Since CaMK2A can be activated by a calcium surge, we further investigated whether hypoxia‐induced oxidative stress activated CaMK2A through increasing intracellular calcium. Results showed that CoCl_2_ treatment induced significant calcium influx and oscillation, which could be abolished by pre‐treating the cells with NAC (Figure 5B–D), indicating hypoxia‐induced oxidative stress increased intracellular calcium level. To further confirm whether hypoxia activates CaMK2A through increasing calcium level, HCC827 cells treated with CoCl_2_ were co‐treated with a calcium chelator BAPTA/AM. BAPTA/AM treatment could abolish CoCl_2_‐induced upregulation of pCaMK2A and GSTP1 levels (Figure 5E), suggesting that hypoxia induced CaMK2A phosphorylation through stimulating oxidative stress and increasing intracellular calcium level.

We next investigated whether the CaMK2A/GSTP1 axis acts as regulatory mechanism to maintain the ROS homeostasis of CSCs. CaMK2A or GSTP1 silencing led to significantly increased intracellular ROS in HCC827 and H1975 cells, whereas CaMK2A or GSTP1 overexpression in A549 cells suppressed intracellular ROS levels detected by both CellROX Deep Red probe and H2DCFDA dye (Figure 5F–I; Figure S7A–C, Supporting Information). Furthermore, we investigated the role of CaMK2A/GSTP1 in regulating mitochondrial ROS using the mitoSOX dye. Downregulation of CaMK2A or GSTP1 in HCC827 cells significantly boosted the mitochondrial ROS levels, while upregulation of CaMK2A released mitochondrial ROS stress in A549 and H1299 cells (Figure 5J,K; Figure S7D, Supporting Information). Concurrent CaMK2A‐OE and GSTP1‐KD compromised both intracellular and mitochondrial ROS suppression, indicating CaMK2A diminished ROS through GSTP1 (Figure 5L,M; Figure S7E, Supporting Information). We speculated that GSTP1‐KD suppresses CSC phenotypes through perturbing the ROS balance of CSCs, which might be rescued through by adding NAC. Results showed cells with GSTP1 suppression displayed impaired ability to form tumorspheres and reduced drug resistance to cisplatin and gefitinib; however, treatment with NAC restored these capabilities in a dose‐dependent manner (Figure 5N–P). Similar results were observed in H1975 cells with GSTP1‐KD and NAC treatment (Figure S7F,G, Supporting Information). NAC treatment alone did not alter GSTP1 level in either control or GSTP1‐KD cells, indicating NAC rescued CSC phenotypes by directly affecting ROS rather than affecting the expression of GSTP1 (Figure S7J, Supporting Information). Together, these results demonstrated CaMK2A/GSTP1 axis supported CSC properties through maintaining redox homeostasis.

### 2.8 GSTP1 was Upregulated and Correlated with Poor Prognosis of Human LUAD

**Figure 6:**
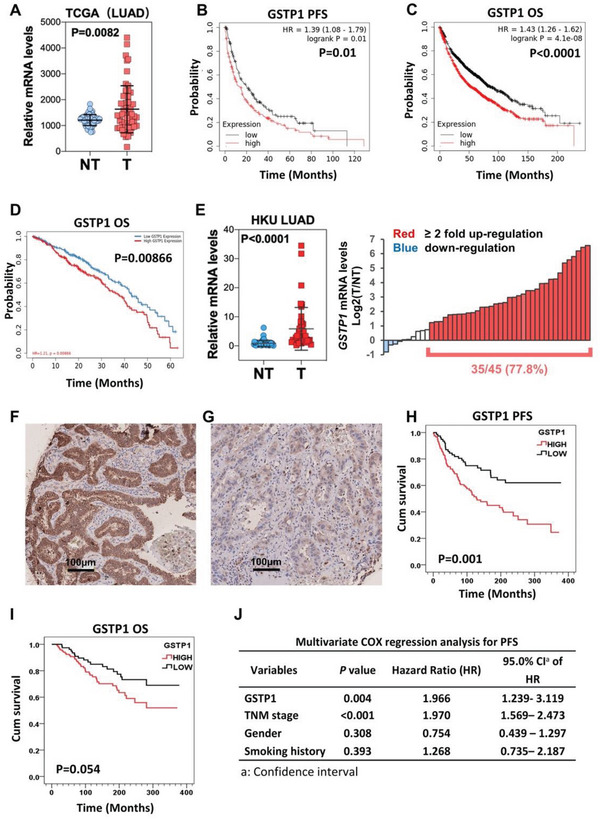
GSTP1 was upregulated and correlated with poor prognosis of human LUAD. A) Scatter plot of *GSTP1* relative mRNA levels in 58 paired LUAD (T) and corresponding non‐tumor (NT) lung from TCGA database. B,C) Kaplan–Meier curves comparing progression free (PFS)sh (B) and overall survival (OS) (C) of GSTP1 expressions stratified by median level, based on the Kaplan–Meier Plotter database (http://kmplot.com/analysis/index.php?p=service&cancer=lung). D) Kaplan–Meier curves comparing 5‐year overall survival (OS) stratified by GSTP1 expressions analyzed by the TIMER2.0 database (http://timer.cistrome.org). E) Scatter dot (left) and waterfall (right) plot of *GSTP1* mRNA levels in paired LUAD and non‐tumor (NT) lung of 45 local patients. F,G) Immunohistochemistry (IHC) images of high (F) and low (G) GSTP1 expression (brown staining) in LUAD. H,I) Kaplan–Meier curves and log‐rank tests of PFS (H), or OS (I), stratified by GSTP1 IHC expression categories in 197 LUAD of local patients. J) Cox regression analysis of PFS using GSTP1 expression level, pathological stage, gender, and smoking history as variables.

The clinical significance of GSTP1 was evaluated. Analysis of 15 GST isoforms of human lung cancer using the TCGA dataset showed *GSTP1*, *GSTA2*, *GSTA5*, *GSTO2*, and *GSTZ1* were significantly upregulated in LUAD compared to corresponding normal tissues, suggesting their potential oncogenic effects on LUAD (**Figure** **6**A; Figure S8, Supporting Information). Amongst these five up‐relegated genes, *GSTP1* was the only isoform that correlated with both shorter progression‐free (PFS, *p* = 0.01) and overall survivals (OS, *p* < 0.0001), using the online public Kaplan–Meier Plotter database (http://kmplot.com/analysis/index.php?p=service&cancer=lung) (Figure 6B,C; Figure S9A,B, Supporting Information). Lung cancer patients with tumors showing higher expression of GSTP1 also exhibited lower 5 years overall survival rate according to the additional online public database TIMER2.0 (http://timer.cistrome.org) (Figure 6D). Consistently, results from local primary LUAD patients showed normalized *GSTP1* mRNA was significantly upregulated by ≥twofold in 35 of 45 (77.8%) tumors (Figure 6E). Immunohistochemistry revealed 61.4% of 197 LUAD showed high level expression of cytoplasmic GSTP1 (Figure 6F) while the others showed none or low expression (Figure 6G). Log rank tests showed higher GSTP1 expression correlated with both shorter PFS (*p* = 0.001) and OS (*p* = 0.054) (Figure 6H,I). Cox regression analysis further showed GSTP1 expression level was an independent risk factor for shorter PFS of LUAD (Figure 6J).

### 2.9 Patient‐Derived *ALK*‐Translocated LUAD Organoids Showed GSTP1 Is a Potential Therapeutic Target against CSC

**Figure 7:**
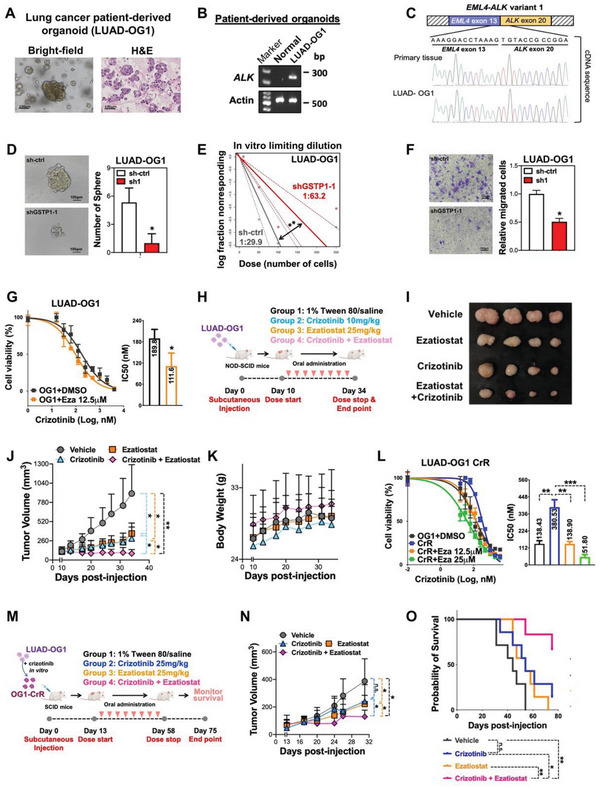
Patient‐derived tumor organoids of *ALK*‐translocated LUAD showed GSTP1 regulated CSC. A) Images of cancer organoids under bright‐field illumination (left) and by H&E stained histology (right). B) Identification of *ALK*‐translocation variant of LUAD‐OG1 by PCR. C) Sequencing results of patient‐derived primary tissue and LUAD‐OG1 verified the *EML4‐ALK* variant 1. D) Tumorsphere formation assay of LUAD‐OG1 with or without GSTP1‐KD. Representative bright field images of tumorsphere (left) and histograms of sphere numbers (right) were shown. E) In vitro limiting dilution assay for frequency of CSCs LUAD‐OG1 tumorspheres with or without GSTP1‐KD. CSC frequency was estimated using public ELDA online tool (http://bioinf.wehi.edu.au/software/elda/). F) Transwell migration assay of LUAD‐OG1 with or without GSTP1 downregulation. Histograms showed relative migrated proportion of the respective cells. G) In vitro effects of treatment with the GSTP1 inhibitor ezatiostat on crizotinib sensitivity in LUAD‐OG1 evaluated by Cell titer–Glo cell viability assay. H) Schematic diagram of the treatment regimen with 1% Tween 80/saline (Group 1), crizotinib (10 mg kg^−1^; Group2), ezatiostat (25 mg kg^−1^; Group 3), or the combination of ezatiostat with crizotinib (Group 4) in NOD‐SCID mice. *n* = 4 mice per group. I) Representative image of LUAD‐OG1 xenografts from the four groups at the endpoint are shown. J) Graph of tumor growth curve during 24 days treatment. K) Graph of animal body weight monitored twice a week. L) Ex vivo effects of GSTP1 inhibitor ezatiostat treatment on crizotinib sensitivity in crizotinib‐resistant LUAD‐OG1 evaluated by Cell titer–Glo cell viability assay. M) Schematic diagram of oral gavage of the treatment regimen with 1% Tween 80/saline (Group 1), crizotinib (25 mg kg^−1^; Group2), ezatiostat (25 mg kg^−1^; Group 3), or the combination of ezatiostat with crizotinib (Group 4) in SCID mice. *n* = 6–7 mice per group. N) Graph of tumor growth curve until the first death occurred (Day 31). O) Kaplan–Meier survival curves showing the tumor‐free survival rate of each group. Tumor volume exceeding 500 mm^2^ was considered as human endpoint according to the guideline of animal ethics. * *p* < 0.05, ** *p* < 0.001, *** *p* < 0.005 versus respective control by Student's *t*‐test.

Patient‐derived organoids could recapitulate both the histological and genetic characteristic of the corresponding primary tumor tissue, which serve as an ideal preclinical model for guiding personalized medicine. To validate the role of GSTP1 in CSC maintenance by a more clinically relevant model and explore whether GSTP1 could serve as a therapeutic target in LUAD treatment, we established cancer organoids from a resected primary LUAD with *ALK* translocation (LUAD‐OG1) as model tissues for further in vitro and in vivo studies. IHC of the in LUAD‐OG1 organoids showed ALK was over‐expressed, and the presence of *EML4‐ALK* fusion variant 1 was further validated by RT‐PCR and sanger sequencing using specific fusion primers,^[^ ^25^ ^]^ consistent with the genotype of the parental cancer (**Figure** **7**A–C; Figure S10A, Supporting Information).

The role of GSTP1 in modulating CSC phenotypes of LUAD organoids was first studied. Knockdown of GSTP1 in LUAD‐OG1 significantly suppressed tumorspheres formation, reduced CSC frequency assessed by in vitro limiting dilution assay, and inhibited cell mobility (Figure 7D–F; Figure S10B, Supporting Information), supporting our hypothesis that GSTP1 regulated stemness and CSC properties in LUAD.

Furthermore, we explored the therapeutic potentials of pharmacological inhibition of GSTP1 by using a GSTP1 inhibitor ezatiostat, an FDA‐approved drug for myelodysplastic syndromes. In vitro, combined treatment of LUAD‐OG1 using crizotinib and 12.5 µm of ezatiostat significantly enhanced the sensitivity of LUAD‐OG1 to crizotinib (Figure 7G). In vivo, treatment of LUAD‐OG1 xenografts in NOD‐SCID mice with either crizotinib or ezatiostat alone led to significant and comparable magnitudes of tumor inhibition compared to vehicle control, but combined treatment resulted in significantly enhanced tumor suppression (Figure 7H–J). Notably, both single treatment and combined treatment were well‐tolerated with maintenance of the body weight of treated mice (Figure 7K). Since drug resistance is attributed to CSC, and development of acquired resistance is inevitable in patients undergoing TKI treatment, we further investigated the therapeutic potential of ezatiostat in combination treatment with crizotinib in tumors with acquired crizotinib resistance. To mimic the clinical circumstance, crizotinib‐resistant (CrR) organoids (LUAD‐OG1 CrR) were established by long‐term treatment of LUAD‐OG1 with increasing doses of crizotinib. In vitro drug sensitivity assay showed that the IC_50_ of crizotinib displayed 1.75‐fold increase in LUAD‐OG1 CrR compared to parental organoids, which could be significantly reversed by addition of ezatiostat in a dose‐dependent manner (Figure 7L). In vivo, compared with the vehicle group, crizotinib treatment of LUAD‐OG1 CrR xenografts in severe combined immunodeficiency (SCID) mice showed non‐significant differences in tumor growth, whereas combined treatment with ezatiostat significantly inhibited tumor growth and prolonged survival, without significantly affecting the body weight of treated mice (Figure 7L–N; Figure S10C,D, Supporting Information). Together, these results indicated GSTP1 was a promising pharmacological treatment target in human LUAD with great potential in targeting CSC phenotypes and reversing acquired drug resistance.

## 3 Discussion

Ubiquitously expressed GSTs mediate detoxification through glutathione conjugation which accounts for their well‐known role in drug elimination and resistance to cancer chemotherapy. We observed amongst 15 GST isoenzymes, GSTP1 was the only upregulated entity in clinical lung cancers that correlated with adverse patient outcomes. In a panel of 10 LUAD cell lines, we found that GSTP1 levels were significantly and positively correlated with CSC markers expression. CSC is crucial for control of cancer perpetuation, propagation, and therapeutic resistance. GSTP1 has been recently reported to be upregulated in lung CSCs and contributes to cisplatin resistance.^[^ ^26^ ^]^ GSTP1 detoxifies cisplatin by two mechanisms: one is by catalyzing the conjugation between cisplatin and glutathione, which will be further transported out of cells;^[^ ^27^ ^]^ the other is by direct sequestering and inactivating cisplatin without affecting its enzyme activity.^[^ ^28^ ^]^ Therefore, its function in mediating cisplatin‐resistance in lung cancer cells, as well as its upregulation in cisplatin‐induced lung CSCs is expected.^[^ ^12^ ^]^ However, whether GSTP1 contributes to CSC phenotypes, resistance to targeted therapy and the corresponding regulatory mechanisms in LUAD remain unknown. If GSTP1 could be shown to play a role in sustaining CSC phenotypes and the molecular pathways involved could be uncovered, it might serve as a useful therapeutic target for lung cancer.

In this study, we utilized a variety of cell models that harbored common driver mutations in lung cancer including *KRAS* substitution (A549) and *EGFR* activation mutations (HCC827, H1975). Importantly, we also freshly raised cancer organoids which are reported as useful models for predicting patient‐specific drug responses,^[^ ^29^ ^]^ from a resected primary LUAD with *ALK* translocation (LUAD‐OG1). Moreover, through GSTP1 up‐ or downregulation, we showed GSTP1 mediates in vitro and in vivo CSC capacities, including stemness, cell mobility, tumorigenicity, metastases, and drug resistances to both cytotoxic and targeted agents. Our study provided extensive evidences on the prominent functions of GSTP1 on maintenance of CSC pool and regulation of CSC properties, providing support to our hypothesis that GSTP1 is a highly important candidate of CSC regulation.

We have previously reported CSCs are positively regulated by the calcium pathway mediator CaMK2A which causes EZH2^T487^ phosphorylation and epigenetic de‐repression of SOX2, conferring CSC phenotypes likely due to re‐programming of the pluripotency status of involved cells.^[^ ^18b^ ^]^ Meanwhile, GSTP1 is a known transcriptional target of the important oxidative stress response factor NRF2. Thus, we hypothesized CaMK2A, NRF2, and GSTP1 might form a regulatory axis and participate in maintaining the CSC population. Using Co‐IP, dual luciferase reporter assay, and in vitro kinase assay, we demonstrated CaMK2A phosphorylates NRF2 at S558, promoting NRF2 nuclear accumulation, which in turn regulates transcriptional activities from specific NRF2 binding sites on *GSTP1*. Notably, our data showed that *GSTP1* but not the other four known downstream targets of NRF2 (*GCLC*, *HOMX1*, *NQO1*, and *TRXR1*) was the downstream regulator of CaMK2A/NRF2 axis, suggesting trans‐regulatory role of CaMK2A/NRF2 S558 in *GSTP1* might be gene‐specific. This hypothesis could be supported by the evidences that phosphorylation of NRF2 at S374, 408, and 433 by AMPK displayed specific regulatory effects on *HOMX1* instead of *GCLC* and *NQO1* expression.^[^ ^30^ ^]^ Besides, consistent with our data that *NQO1* was not the downstream target of CaMK2A/NRF2 S558 axis, Danna D. Zhang's group pointed out that phosphorylation of NRF2 at S558 by MAPKs exhibited limited effects on regulating *NQO1* and *GSTA1* expression in MDA‐MB‐231 breast cancer cells.^[^ ^31^ ^]^ Since they did not study the transcriptional activity of NRF2 on *GSTP1* binding sites in MDA‐MB‐231, whether this transactivation was cancer type‐dependent is still needed to be further explored. As for the mechanisms of NRF2 S558 on facilitating GSTP1 expression, apart from current findings that NRF2 S558 promoted its nuclear translocation, phosphorylation at S558 might regulate transcriptional activity by changing conformational interaction with regulator proteins and/or modulating binding efficiency toward promoter and/or enhancer, since NRF2 S558 resides in the CNC‐basic leucine zipper domain is involved in NRF2 binding to conserved antioxidant response elements.^[^ ^32^ ^]^

Although we have provided solid evidence that CaMK2A regulates GSTP1 through NRF2, correlation analysis of the expression level of pCaMK2A/nuclear NRF2 and GSTP1 did not show NRF2 was correlated with either GSTP1 or pCaMK2A in both clinical lung cancer or lung cancer cell lines. Notably, the most well‐known activation mechanism of NRF2 relies on its release from KEAP1,^[^ ^33^ ^]^ suggesting conventional activation of NRF2 might be related to KEAP1 mutation status. Accordingly, we showed A549 and H1648, which are KEAP1 mutant cell lines, displayed relatively higher level of NRF2, compared to the KEAP1 wild‐type cell lines including H1299 and HCC827 (Figure S4J, Supporting Information). However, the expressions of pCaMK2A and GSTP1 were positively correlated in both clinical lung cancer samples and lung cancer cell lines, indicating CaMK2A/NRF2 S558 could regulate GSTP1 in a KEAP‐independent manner. This result is also consistent with the findings that overexpression of KEAP1 completely suppressed the expression of GSTA1 instead of GSTP1 in Caco‐2 colorectal adenocarcinoma cells.^[^ ^34^ ^]^ Together, results from current study indicated that the CaMK2A/NRF2/GSTP1 comprises a functional axis in CSC regulation.

In lung cancer, homeostatic disequilibrium due to metabolic and hypoxic stresses is the chief stimuli for CSC phenotypes. In particular, low intracellular ROS levels are required to maintain stem‐like cells in both normal tissues and cancers.^[^ ^35^ ^]^ Hypoxia has been reported to enrich CD166^+^ and CD133^+^ stem‐like cancer cell populations.^[^ ^5a,c^ ^]^ Consistently, we also observed hypoxia stimulated the CSC markers ALDH1 and CD44V8‐10. Specifically, we found that GSTP1 supported CSC properties through regulating CD44V8‐10 and was not other variants. In lung cancer, CD44V8‐10 has been reported to enrich stem‐like cells,^[^ ^36^ ^]^ suggesting CD44V8‐10 could serve as a CSC marker in lung cancer. Due to the fact that CD44V8‐10 can regulate redox system through promoting glutathione synthesis,^[^ ^37^ ^]^ it is possible that GSTP1 regulated CD44V8‐10 expression based on positive feedback mechanism related to glutathione depletion. In this study, we showed an in vitro hypoxic environment activated the CaMK2A/NFR2/GSTP1 axis through oxidative stress induced calcium influx. In vivo, co‐regulated CaMK2A and GSTP1 levels also corresponded with regions with differential hypoxia levels designated by CA9 expression. Dynamic manipulation experiments showed this axis prevented ROS accumulation and enhanced resistance of tumor cells to chemotherapy and targeted therapy. In the literature, upregulation of activated CaMK2 under hypoxia has been reported in liver cancer but details regarding the isoform CaMK2A were not provided.^[^ ^38^ ^]^ Apart from calcium‐dependent activation demonstrated by the current work, studies on CaMK2A have shown hypoxia causes oxidation at C280/M281 leading to T286 autophosphorylation and CaMK2A activation in a calcium‐independent manner,^[^ ^39^ ^]^ suggesting hypoxia might directly activate CaMK2A.

Our findings indicate GSTP1 to be a potential therapeutic target of lung cancer. Evidences from genetic manipulation studies show GSTP1 facilitates cell viability and increases resistance to cisplatin and targeted therapy including gefitinib, erlotinib, or afatinib for the relevant *EGFR* mutated, and crizotinib for *ALK* translocated cancers. Importantly, we tested the GSTP1 specific pharmacologic inhibitor ezatiostat on freshly raised cancer organoids and induced crizotinib‐resistant organoids bearing ALK fusion proteins. Ezatiostat is a tripeptide glutathione analog with isoform‐specific inhibitory action on GSTP1 catalytic activity, and has been used in pre‐clinical models and clinical studies of patients with myelodysplasia to stimulate normal myeloblasts proliferation.^[^ ^40^ ^]^ Our study showed in organoid‐derived xenografts, ezatiostat alone led to significant tumor reduction at comparable extent and tempo as crizotinib treatment, while co‐treatment resulted in significantly augmented tumor shrinkage. More importantly, ezatiostat significantly sensitized the organoids induced for crizotinib resistance both in vitro and in vivo, with significantly prolonged overall survival, compared to single treatment alone. Notably, there were no signs of drug toxicity in mice treated with ezatiostat in single or combined therapy. Thus, as an FDA‐approved drug, ezatiostat could be a potential agent for combination treatment of lung cancer. Further testing on a larger spectrum of lung cancers would be required to confirm this postulation.

**Figure 8:**
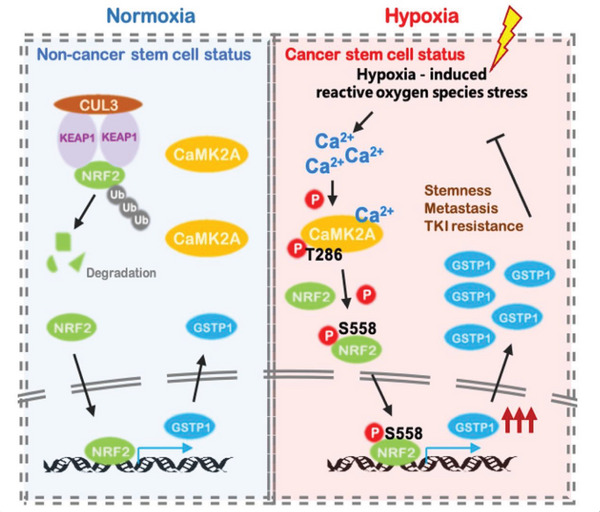
Schematic depiction of molecular mechanism of CSC regulation through hypoxia‐induced CaMK2A/NRF2/GSTP1 pathway. Hypoxia induced ROS stress triggers the increase of intracellular calcium level, and further activates CaMK2A by phosphorylation at T286. CaMK2A activation leads to an upregulated GSTP1 via direct phosphorylation of NRF2 at S558 residue in a KEAP1 independent manner. In CSC where the CaMK2A/NRF2/GSTP1 axis is activated, ROS homeostasis is maintained by GSTP1 upregulation, enabling self‐renewal, cell survival, drug resistance, tumor longevity, and propagation.

In summary, we showed hypoxia‐induced CaMK2A activation leads to GSTP1 upregulation through direct NRF2 phosphorylation at S558, in turn eliminating ROS and facilitating homeostatic equilibrium through negative feedback, eventually contributing to lung cancer CSC maintenance (**Figure** **8**). Our study provided novel data to show CaMK2A/NRF2 S558/GSTP1 as a functional and targetable axis supporting CSC maintenance. Furthermore, we identified GSTP1 as a promising treatment target of LUAD not only through evidences of in vitro and in vivo GSTP1‐KD studies, but also by in vivo results of an *ALK*‐translocated cancer organoid model employing the FDA‐approved drug ezatiostat as a single agent or in combination with targeted therapy.

## 4 Experimental Section

### Cell Lines and Patient Samples

Established human NSCLC cell lines (A549, H1299, H1975, H441, HCC827, H820, H1819, and H1648) and HEK293T were purchased from ATCC. Patient‐derived cell lines (HKULC4, PDCL#24, and FA10) were established from resected primary lung cancers or malignant pleural effusion. Cells were cultured in RPMI1640 (Invitrogen, Carlsbad, CA) with 10% FBS. All procured cell lines used in this study were authenticated using the AmpFlSTR Identifiler PCR Amplification Kit for short tandem repeat profiling according to the manufacturer's instruction (Thermo Fisher Scientific, Waltham, MA). Primary lung cancer and corresponding normal lung tissues were obtained from surgical specimens of ethnic Chinese collected in Queen Mary Hospital, Hong Kong. Tissue collection protocols were approved by the HKU/HA HKW Institutional Review Board, the assigned study number is UW10‐168. Tissues were sampled after written informed consents were obtained from patients. Tumor diagnosis and staging were assessed by a qualified pathologist (MPW).

### Tumor Microarray and Immunohistochemistry

At least two tumor cores from each case were selected and assembled into tissue microarray blocks (TMA). IHC was performed according to routine procedures after antigen retrieval by scientific microwave at 95 °C for 15 min in pH 8.0 EDTA. Primary antibodies of GSTP1 (1:2000; Sigma #HPA019869) were incubated overnight at 4 °C, followed by secondary antibodies conjugated with polymer‐linked HRP (DAKO, Agilent) for 30 min at room temperature. ALK IHC was stained by anti‐ALK (D5F3) CDx assay (Ventana). Expression levels of GSTP1 and pCaMK2A T286 were scored according to the extent and intensity of cytoplasmic staining in the tumor cells only. The intensity was graded as 1, 2, or 3 according to whether cytoplasmic staining was absent or weak, moderate, or strong, respectively. The staining extent was graded as 1, 2, or 3 according to whether expression was observed in scattered individual cells, aggregates of ≥5 but ≤100 cells, or sheets of >100 cells. The products of the two grades were then computed, and cases with scores of ≥4 were counted as high level expression.

### Plasmids

shRNAs targeting human GSTP1 and CaMK2A were purchased from Sigma‐Aldrich (St Louis, MO). The PCDH‐CMV‐MCS‐EF1‐COPRFP vector (SBI, Mountain View, CA) was used for the construction of stable GSTP1 and CaMK2A overexpression vector by inserting the full‐length targets. The coding sequence of GSTP1 was amplified from total cDNA of H1975 cell line. The plasmid used for the overexpression of NRF2 (pCDNA3‐Myc3‐Nrf2) was purchased from the Addgene (Cambridge, MA; Plasmid #21 555). Site directed mutagenesis of NRF2 T267A, NRF2 S558A, and NRF2 T586A were performed using QuickChange (Stratagene, La Jolla, CA) according to manufacturer's instructions. Luciferase vectors and pLenti6/V5 TOPO‐NRF2, NRF2 S558A, and NRF2 S558D vectors were purchased from GeneScript (NJ, USA). The primers used in this study were listed in Table S1, Supporting Information.

### Quantitative Real‐Time PCR

RNA was isolated from monolayer cells by using RNAiso Plus reagent (Takara, Mountain View, CA). Complementary DNA was synthesized from total RNA using PrimeScript RT Reagent Kit (Takara, Mountain View, CA). Transcript levels were analyzed using SYBR green (Qiagen, Hilden, Germany) and qPCR programs were run on a 7900HT FAST Real‐time PCR system (Applied Biosystems, Carlsbad, CA). The levels of *beta‐macroglobulin (B2M)* and *RPL13A* of each test sample were respectively assessed and the averaged level was used as the internal control. Primers used were listed in Table S1, Supporting Information. Relative gene expression levels were analyzed using the 2^−△△Ct^ method.^[^ ^41^ ^]^

### Detection of *EML4‐ALK* Fusion Variant by Reverse Transcriptase‐Polymerase Chain Reaction and Sanger Sequencing

Total RNA of primary lung cancer tissue and LUAD‐OG1 was extracted and used for ALK variant identification. The primers Fusion‐TR‐S (5′‐GTGCAGTGTTTAGCATTCTTGGGG‐3′) and Fusion‐RT‐AS (5′‐TCTTGCCAGCAAAGCAGTAGTTGG‐3′) were used to detection the *EML4‐ALK* fusion cDNA,^[^ ^42^ ^]^ and RT‐PCR was performed with denaturation at 95 °C for 4 min, followed by 40 cycles of amplification stage including three steps −95 °C for 30 s, 55 °C for 30 s, and 72 °C for 150 s, and extension at 72 °C for 10 min.^[^ ^43^ ^]^ RT‐PCR for *β*‐actin was used as control for RNA quality. PCR products were further used for sanger sequencing with the BigDye Terminator version 3.1 cycle sequencing kit (Applied biosystems) according to the manufacturer's instructions.

### Sphere Formation Assay and Serial Passage

For sphere formation assays, 250 cells of A549, 500 of HCC827, and 1000 of H1975 were seeded in low‐attachment plate (Costar) with cancer stem cell (CSC) medium (RPMI1640 with 20 ng mL^−1^ EGF, 20 ng mL^−1^ FGF, 40 ng mL^−1^ IGF, and 1 × B27) for 10 days. Harvested spheres were dissociated by trypsin and re‐seeded using the same settings as the first generation.

### Migration and Invasion Assay

Corning Transwell was used for migration and invasion assays. For a migration assay, 5 × 10^5^ cells were seeded into the upper chambers with serum‐free RPMI1640 medium. Medium with 10% FBS was added to the lower chambers. Cells were allowed to migrate for 24 h. For the invasion assay, 50 µL of 10 × diluted Matrigel was pre‐coated on the upper surface of the permeable membrane and incubated at 37 °C for 30 min. Then cells were seeded into the upper chambers with serum‐free RPMI1640, and RPMI1640 with 10% FBS was added to the lower chambers. Cells were allowed to invade for 48 h. Cells that migrated or invaded to the lower surface of the membrane were fixed with methanol and stained by crystal violet, which were then dissolved by 50 µL of 10% acetic acid and transferred to a 96‐well plate. The dye intensity was measured by plate spectrophotometer at 570 nm.

### Cell Viability Assay

For lung cancer cell lines, cells were seeded in 96‐well plates and incubated at 37 °C for 24 h. Then cells were incubated with 200 µL of medium containing increasing doses of the drugs gefitinib (Selleckchem Houston, TX), erlotinib (Selleckchem Houston, TX), afatinib (Selleckchem Houston, TX), crizotinib (Selleckchem Houston, TX), or cisplatin (Sigma‐Aldrich, St Louis, MO) for 72 h. To detect and calculate the half maximal inhibitory concentration (IC_50_), 5 mg mL^−1^ of (MTT) (Sigma‐Aldrich, St Louis, MO) was added and the mixture was incubated at 37 °C for 4 h. The absorbance was determined at 570 nm. For organoids assays, organoids were dissociated by mechanical shearing, strained through a 70 µm filter, resuspended using LOM medium containing 5% GFR‐BME, and finally 30 µL of suspension were seeded into 384‐well plate. Organoids were treated with increasing concentrations of drugs for 5 days. Cell viability was detected by Cell titer–Glo 2.0 assay kit (Promega) and luminescence were measured by a multifunctional reader. The drug response curve was plotted and IC_50_ was calculated using nonlinear regression model by GraphPad Prism 7.0.

### BrdU Proliferation Assay

Cellular proliferation rate was evaluated by using BrdU cell proliferation kit (Cell Signaling, Beverly, MA) conducted as previously described.^[^ ^5b^ ^]^

### Senescence *β*‐Galactosidase Staining Assay

Cellular senescence was measured by using senescence *β*‐Galactosidase staining kit (Beyotime, China) according to the instruction. Briefly, 100 000 of suspended cells with or without GSTP1‐KD were fixed in 1 mL of fixation at room temperature for 15 min. Then were washed by PBS for three times and stained by 1 mL of staining C solution with 10 µL of solution A, 10 µL of solution B, and 50 µL of X‐Gal solution at 37 °C for overnight. Then the cells were resuspended by 1 mL of PBS and 50 µL of cell suspension was added to the well of the 96‐well plate. Each well randomly captured 2–3 fields and the total cell number and positive cell number were counted by image J.

### In Vitro Kinase Assay

In vitro CaMK2A Kinase assay was performed using CaMK2A kinase assay kit (Promega) according to manufacturer's instruction. Briefly, wild type and mutant Nrf2 proteins were precipitated by 2 µg of myc tag antibody from 500 µg of cell lysates of 293 cells transfected with empty vector control, wild type myc‐Nrf2, myc‐Nrf2 T267A, myc‐Nrf2 S558A, and myc‐Nrf2 T586A, respectively. CaMK2A kinase reaction was conducted on the precipitated Nrf2 proteins in the presence of 10 ng CaKM2A and 25 µm ATP in CaMK2A kinase buffer (40 mm Tris, pH 7.5, 20 mm MgCl_2_, 0.1 mg mL^−1^ BSA, 50 µm DTT and Ca^2+^/Calmodulin solution (0.03 µg µL^−1^ Calmodulin, 1 mm Tris, pH 7.3, 0.5 mm CaCl_2_)) for the indicated time. Kinase activity was measured by ADP‐Glo Kinase Assay kit (Promega) according to the manufacturer's instruction.

### Flow Cytometry and Fluorescence Activated Cell Sorting

ALDH activity was detected by ALDEFLUOR kit (Stem Cell Technologies) according to the manufacturer's instructions. CD44 expression was stained by anti‐CD44‐APC antibody (BD Biosciences) as previously described.^[^ ^15a^ ^]^ Cells were then analyzed using FACS Canto II (BD Biosciences). Results were analyzed using FlowJo software (Tree star). Cell isolation and enrichment were performed using BD Aria sorter (BD Biosciences). The purity of sorted cells was at least 95%.

### Redox Oxidative Species Detection Assay

Intracellular ROS levels were detected by CellROX Deep Red probe (Life Technologies) and H2DCFDA (Invitrogen). Mitochondrial ROS levels were detected by mitoSOX Red Mitochondrial Superoxide indicator (Invitrogen) according manufacturer's instructions, respectively. Fluorescent signals were detected using FACS Canto II analyzer (CellROX by APC channel; H2DCFDA by FITC channel; mitoSOX by PE channel) and data analysis was performed using FlowJo.

### Annexin V Apoptosis Staining Assay

Apoptosis rate was evaluated by Annexin V and PI co‐staining using FITC Annexin V Apoptosis Detection Kit I (BD Biosciences) according to the manufacturer's instructions. Briefly, 1 × 10^5^ cells were resuspended in 100 µL of 1X binding buffer and stained with 5 µL of FITC Annexin V and 5 µL PI for 15 min at room temperature. Apoptotic rates were analyzed by FACS Canto II analyzer.

### Dual Luciferase Reporter Assay

Cells were transfected with expression plasmids pRL‐TK and reporter plasmids using lipofectamine 2000 and cultured at 37 °C for 48 h. Luciferase activities were then measured by the Dual‐Luciferase Reporter Assay System (Promega) and Dual‐Glo Luciferase Assay System (Promega) according to manufacturer's instructions. The luminescent signals emitted from firefly and renilla luciferases were recorded by a multifunctional reader.

### Co‐Immunoprecipitation Assay and Western Blot Analysis

Cells transfected with expression vectors were collected and lysed by NETN buffer (25 mm Tris‐HCl pH 8.0, 50 mm NaCl, 0.2 mm EDTA, and 0.1% NP40) with freshly added 1:50 Phosphatase Inhibitor Cocktail 2 (Sigma), 0.1 M DTT (Thermo Fisher Scientific) and sodium fluoride (New England BioLabs). Then 1500 mg of cell lysate was incubated with 3 µg of anti‐CaMK2A (Santa Cruz, CA) antibody with gentle rotation at 4 °C for 4 h. The antibody complex was incubated with pre‐washed protein‐G beads (protein‐G Mag Sepharose Xtra, GE Healthcare) at 4 °C overnight. The complex was washed with NETN buffer five times and eluted. The immunoprecipitants were subjected to western blot analysis conducted as previously described.^[^ ^5b^ ^]^

To mimic the hypoxic condition, cells were incubated under low oxygen (1% O_2_, 5% CO_2_, and 94% N_2_) in hypoxia chamber for 24 h; or cells were treated with 100 µm of cobaltous chloride (CoCl_2_) for 24 h. The primary antibodies included GSTP1 (1:1000; Abcam #ab13849), phospho‐CaMK2A T286 (1:500; Santa Cruz #sc12886‐R), CaMK2A (1:500; Santa Cruz #sc13141), NRF2 (1:1000; Abcam #ab62352), HIF1A (1:1000; Abbexa #abx033664), *β*‐actin (1:2000; Cell signaling #4970s), E‐cadherin (1:1000; Cell signaling #4065) and VIMENTIN (1:1000; Cell signaling #3932s), TBP (1:2000; Cell signaling #8515s), KEAP1 (1:1000; Santa Cruz #sc365626), and *β*‐tubulin (1:2000; Invitrogen #PA5‐16863).

### Intracellular Calcium Measurement

Before calcium measurements, cells were washed with Margo's solution. Then cells were loaded with 3 µm Fura‐2‐AM (Life Technologies, USA) and 1 µm Pluronic F‐127 (Life Technologies, USA) in the Margo's solution at 37 °C for 30 min before being washed with the Margo's solution and stabilized in the solution for another 30 min with or without the calcium inhibitor at 37  °C. The coverslip was transferred to a mini chamber containing 1 mL Margo's solution and mounted on to a fluorescence microscope (Eclipse Ti, Nikon, Tokyo, Japan). Fluorescence was alternatively excited by dual wave length at 340 and 380 nm, and emission signals were recorded at 510 nm. Intracellular calcium change was reflected by the change in the ratio of 340/380 fluorescent signal intensity.

### Immunofluorescent Staining

Cells were pre‐seeded onto coverslip 1 day before fixation. Then cells were washed three times by PBS and fixed by 4% PFA at room temperature for 15 min. Then washed the cells for three times by PBS and treated with 0.3% Triton‐X at room temperature for 10 min, followed by 5% BSA blocking for 1 h at room temperature. Primary antibody included NRF2 (1:200; Abcam #ab62352) and Myc‐Tag (1:500, Cell signaling, #2276s) were added to the cells and incubated for overnight at 4 °C. After incubation, cells were washed by PBS for three times and incubated with secondary antibody for 1 h at room temperature, followed by staining of Hoechst 33 342 (1:1000, Thermo Scientific, #62 249) at room temperature for 20 min. Then the cells were washed with PBS and the coverslip was mounted onto slide. The fluorescent signal was detected by fluorescence microscope (Eclipse Ti, Nikon Tokyo, Japan).

### Multiplex Fluorescent IHC Staining

Multiplex immunofluorescent staining was performed using Opal Immunology Discovery kit (Akoya biosciences) on formalin fixed paraffin embedded tissues according to the manufacturer's instructions. Briefly, sections were deparaffinized using xylene and hydrated using gradient ethanol ending with a distilled water wash. Microwave treatment was performed in AR6 buffer for antigen retrieval. Then slides were incubated with a primary antibody followed by secondary antibody. Opal fluorophore solution was added to the slide and incubated for 10 min at room temperature. Microwave treatment was performed again to remove the first primary antibody. Steps were repeated for subsequent primary antibodies to achieve multiplex IF staining. After antibody staining, slides were incubated in DAPI solution for 5 min at room temperature, washed several times, and then mounted with coverslip. The IF signals were detected by Vectra Polaris Imaging System (PerkinElmer) and analyzed by inForm software (Akoya).

### In Vivo Subcutaneous Xenograft Assay

All animal experiments were strictly carried out according to guidelines approved by the Animal Ethics Committee, The University of Hong Kong. The approval numbers of studies are 4020‐16, 5246‐19, and 4631‐18. The designated numbers of cells were suspended in 50 µL of cold RPMI1640‐serum free medium. The same volume of Matrigel (BD Pharmingen) was added to the cell suspension and mixed gently. The cell suspension was then subcutaneously injected into the flanks of 4 weeks‐old SCID mice. Tumor volumes were measured twice weekly using calipers. Tumor volume was calculated using the formula [*W* ^2^ × *L*]/2 and expressed in mm^3^.^[^ ^44^ ^]^

### In Vivo Tail Vein Injection Model

Luciferase‐labeled cells were suspended in 150 µL of filtered PBS buffer and injected into 4 weeks‐old nude mice through the tail vein. At the end of experiment, 150 mg kg^−1^ d‐luciferin solution was given to mice through intraperitoneal injection. Bioluminescence was measured using PE IVIS Spectrum. Mice were then sacrificed and lung tissues were harvested for further analysis.

### Establishment of Organoid Culture

Fresh tissue was processed for cell isolation and long‐term expansion as previously described.^[^ ^45^ ^]^ Briefly, fresh solid tissues were minced and digested in lung organoid medium (LOM) (AdDF+++ (Advanced DMEM/F12 with 1% GluaMax, 1% 1 m Hepes and 1% Penicillin/Streptomycin) with 10% Rspol conditional medium, 10% Noggin conditional medium (kind gift from Prof. Hans Clevers), 1 × B27, 1.25 mm *N*‐acetylcysteine, 10 mm Nicotinamide, 5 µm ROCK inhibitor, 500 nM ALK inhibitor, 1 µm p38 MAP kinase inhibitor, 5 ng mL^−1^ FGF‐7, and 20 ng mL^−1^ FGF‐10 containing 1 mg mL^−1^ collagenase (Sigma) on a shaker at 37 °C for 1 h. Digestion was stopped by the addition of FBS. Digested tissue suspensions were sequentially sheared using 10 and 5 mL plastic pipettes. Afterward, the suspensions were sequentially strained over 100 and 70 µm filter and centrifuged at 400 g. The visible red pellet was lysed by using 3 mL of red blood cell lysis buffer and centrifuged again at 400 g. Then the pellet was resuspended using cold growth factor reduced‐basement membrane matrix (GFR‐BME) (Corning) and 40 µL of cell suspension was seeded in a pre‐warmed 24‐well suspension culture plate (Greiner) at 37 °C for 20 min. After gelation, 500 µL of LOM medium was added to each well. Lung cancer organoids were distinguished from normal cystic organoids by their pleomorphic cell morphology.

Crizotinib‐resistant organoids were cultured with stepwise increase of crizotinib, over a period of 3 months and around ten passages to allow the cells to adapt crizotinib toxicity. The stable crizotinib‐resistant organoids were then maintained in normal complete medium for 2 weeks before the bioassays.

### In Vitro Limiting Dilution Assay

Decreasing numbers of cells were seeded into 96‐well low‐attachment plates. Cells were cultured in CSC medium for 14 days. The number of wells containing spheres were recorded and the in vitro CSC frequency was calculated by an online tool http://bioinf.wehi.edu.au/software/elda/ ^[^ ^46^ ^]^

### In Vivo Drug Treatment Assay

Patient‐derived organoids were subcutaneously injected into the back of 4 weeks male NOD/SCID mice. Once the tumors reached ≈110 mm^3^, the mice were randomly separated into four groups: 1) 1% Tween 80/saline; Ezatiostat single agent (MedChemExpress) diluted in 1% Tween 80/saline (25 mg mL^−1^ per every 2 days); 3) Crizotinib single agent diluted in 1% Tween 80/saline (10 mg kg^−1^ day^−1^); 4) Ezatiostat (25 mg mL^−1^ per every 2 days) and Crizotinib (10 mg kg^−1^ day^−1^) combined treatment group. All drugs were treated by oral gavage. The tumor volume and body weight were measured twice weekly. The mice were treated for 24 days before sacrifice, at which point tumors were harvested for further analysis.

Patient‐derived crizotinib‐resistant organoids were subcutaneously injected into the back of 4‐week male SCID mice. Once the tumors reached ≈100 mm^3^, the mice were randomly separated into four groups: 1) 1% Tween 80/saline; Ezatiostat single agent (MedChemExpress) diluted in 1% Tween 80/saline (25 mg mL^−1^ two times per week); (2) Crizotinib single agent diluted in 1% Tween 80/saline (25 mg kg^−1^ five times per week); (3) Ezatiostat (25 mg mL^−1^ two times per week) and Crizotinib (25 mg kg^−1^ five times per week) combined treatment group. All drugs were treated by oral gavage. The tumor volume and body weight were measured twice weekly. The mice were treated for 45 days and kept for another 17 days after treatment withdrawal. Due to the animal welfare, the mice were sacrificed when the tumor volume over 500 mm^3^.

### Statistics

Data were analyzed by SPSS (version 16.0; SPSS Inc., Chicago, IL, USA), GraphPad Prism 7.0 or Excel (Microsoft, Redmond, WA, USA) software packages and shown as mean ± standard deviations (SD.). Differential expression between paired tumor/normal tissues were analyzed by Wilcoxon text. Differences between groups were analyzed by *t* test for continuous variables. Association between GSTP1 expression and overall survival and recurrence‐free survival were analyzed by the Kaplan–Meier method with log‐rank test. Multivariate survival analyses were performed by Cox regression model. Two‐sided *p* values <0.05 were considered as statistically significant.

## Conflict of Interest

The authors declare no conflict of interest.

## Authors’ Contributions

S.Q.W. and J.J.C. contributed equally to this work. Z.J.X. and M.P.W. conceived the project idea and acquired funding support; S.Q.W., Z.J.X., J.J.C., Y.C.J., and Z.N.L. performed the experiments and analyzed the data; Y.H.P. and Y.C.R. contributed to methodology and experimental design, J.W.P.Y. supervised and contributed to experimental designs. S.Q.W., J.W.P.Y., Z.J.X., and M.P.W. drafted and finalized the manuscript. Z.J.X. designed and supervised the overall conduction of the studies.

## Supporting information

Supporting Information

## Data Availability

The data that support the findings of this study are available on request from the corresponding author upon reasonable request.
