# Supplementary material for: Targeting GSTP1 as Therapeutic Strategy against Lung Adenocarcinoma Stemness and Resistance to Tyrosine Kinase Inhibitors
Source: Adv Sci (Weinh). 2023 Jan 29;10(7):2205262. doi: 10.1002/advs.202205262 (PMC9982593; doi:10.1002/advs.202205262)
Supplement: Supplementary file 1 — Supporting Information [file ADVS-10-2205262-s001.pdf]

Supplementary Information

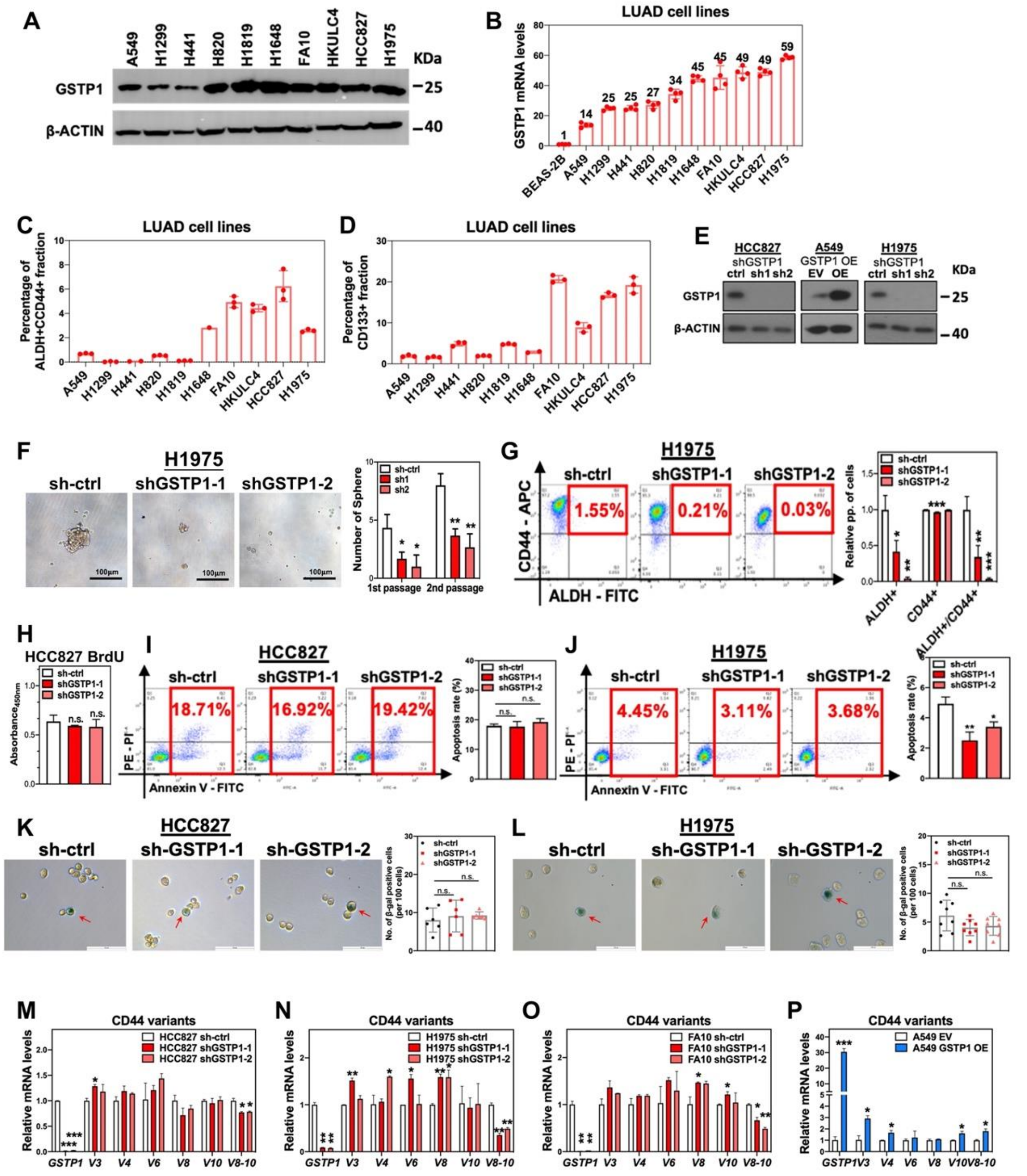

Supplementary figure 1. GSTP1 was up-regulated in CSC and supported stemness in LUAD. (A) Western blot analysis of GSTP1 expression in a LUAD cell line panel. **(B)** *GSTP1* mRNA levels in transformed human bronchial epithelial cells (BEAS-2B) and LUAD cell lines. **(C-D)** Percentages of ALDH^{+}/CD44^{+} **(C)** and CD133^{+} **(D)** CSC population in a LUAD cell line panel detected by flow cytometry. **(E)** Western blot analysis of GSTP1 expression in HCC827, H1975, and A549 cells with or without GSTP1 manipulation. **(F)** Tumorsphere formation in first passage (left), and serial passage assay for two generations (right), of H1975 cells with or without GSTP1-KD. **(G)** Proportions of ALDH^{+}/CD44^{+}

subsets in H1975 cells with or without GSTP1-KD. **(H)** BrdU proliferation assay of HCC827 cells with or without GSTP1-KD. **(I-J)** Apoptosis rate of HCC827 **(I)** and H1975 **(J)** with or without GSTP1-KD exanimated by Annexin V and PI staining. **(K-L)** Senescence rate of HCC827 **(K)** and H1975 **(L)** with or without GSTP1-KD evaluated by **β**-gal staining. **(M-P)** Relative mRNA levels of *CD44* variants in HCC827 **(M)**, H1975 **(N)** and FA10 **(O)** cells with or without GSTP1-KD, and A549 cells with or without GSTP1-OE (**P**). Data represented mean ± SD of triplicate measurements. * p<0.05, ** p<0.001, *** p<0.005 versus respective control by Student's t-test.

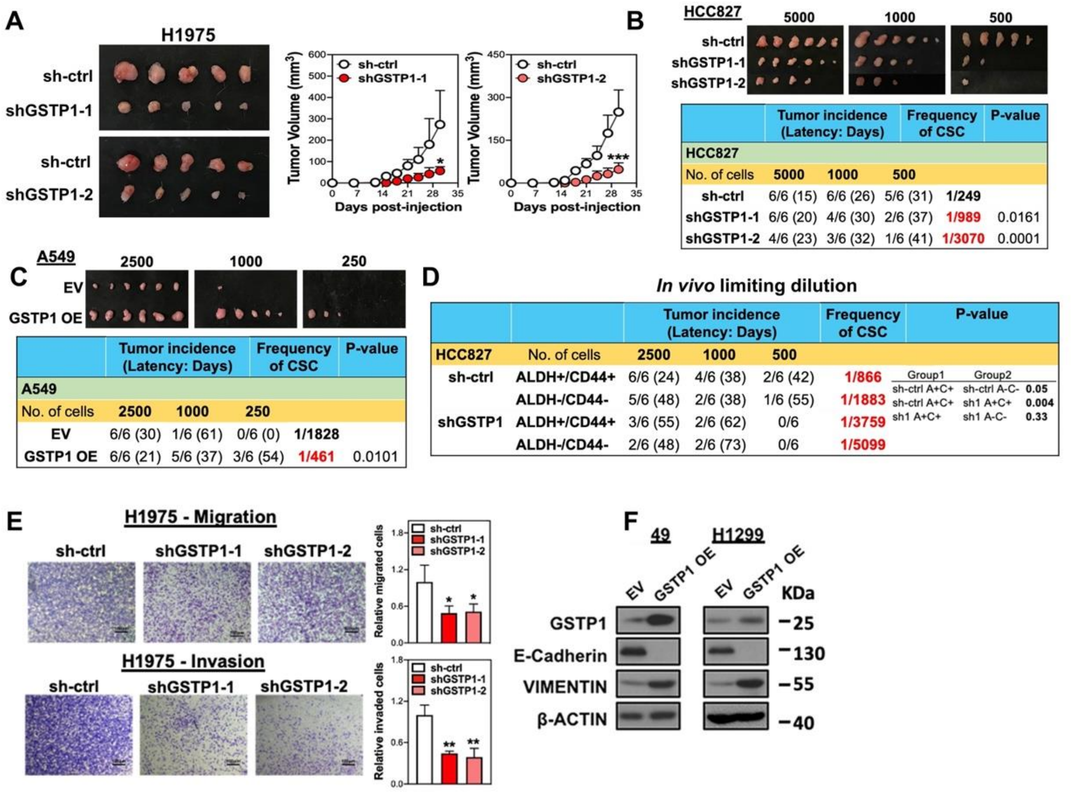

Supplementary figure 2. GSTP1 supported *in vivo* tumorigenicity and migratory and invasive properties in LUAD. (A) Representative images of subcutaneous xenografts of H1975 cells with or without GSTP1 silencing (left), and corresponding tumor growth curves (right). **(B-C)** *In vivo* limiting dilution assay of HCC827 cells with GSTP1-KD **(B)**, and A549 cells with GSTP1-OE **(C)**. Representative images of xenografts (upper panel) and the summarized CSC frequencies calculated by the ELDA online tool (lower panel) are shown. **(D)** *In vivo* limiting dilution assays of $ALDH^+/CD44^+$ and $ALDH^-/CD44^-$ fractions isolated from HCC827 control and GSTP1-KD cells. CSC frequencies and P values were calculated using the ELDA online tool. **(E)** Transwell assays of migration (upper panel), and invasion (lower panel) in H1975 cells with or without GSTP1-KD. Histograms showed the relative migrated and invaded proportions of the respective cells. **(F)** Western blot analysis of

E-cadherin and VIMENTIN expression in A549 and H1299 cells with or without GSTP1-OE. Data is presented as mean ± SD of triplicate measurements. * $p<0.05$, ** $p<0.001$, versus respective control by Student's *t*-test.

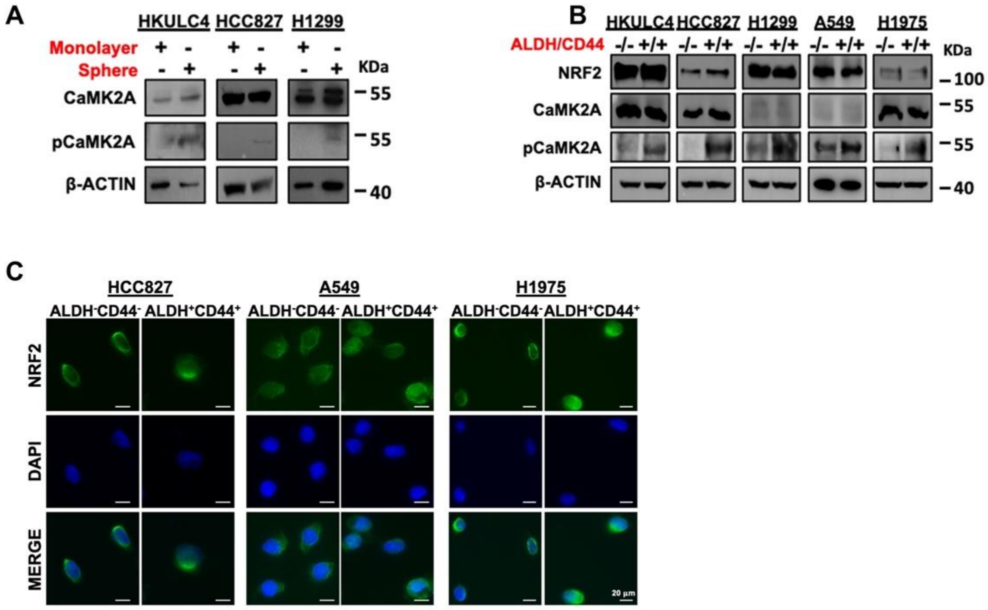

Supplementary figure 3. CaMK2A/NRF2 axis was activated in LUAD CSC subsets. (A) Western blot analysis of CaMK2A and phospho-CaMK2A levels in tumorspheres and corresponding monolayers derived from HKULC4, HCC827 and H1299 cells. **(B)** Western blot analysis of NRF2, CaMK2A and phospho-CaMK2A levels in $ALDH^{+}/CD44^{+}$-CSC and $ALDH^{-}/CD44^{-}$ non-CSC fractions sorted from HKULC4, HCC827, H1299, A549 and H1975 cells, respectively. **(C)** Immunofluorescence staining of NRF2 localization in $ALDH^{+}/CD44^{+}$-CSC and $ALDH^{-}/CD44^{-}$ non-CSC fractions sorted from HCC827, A549 and H1975 cells.

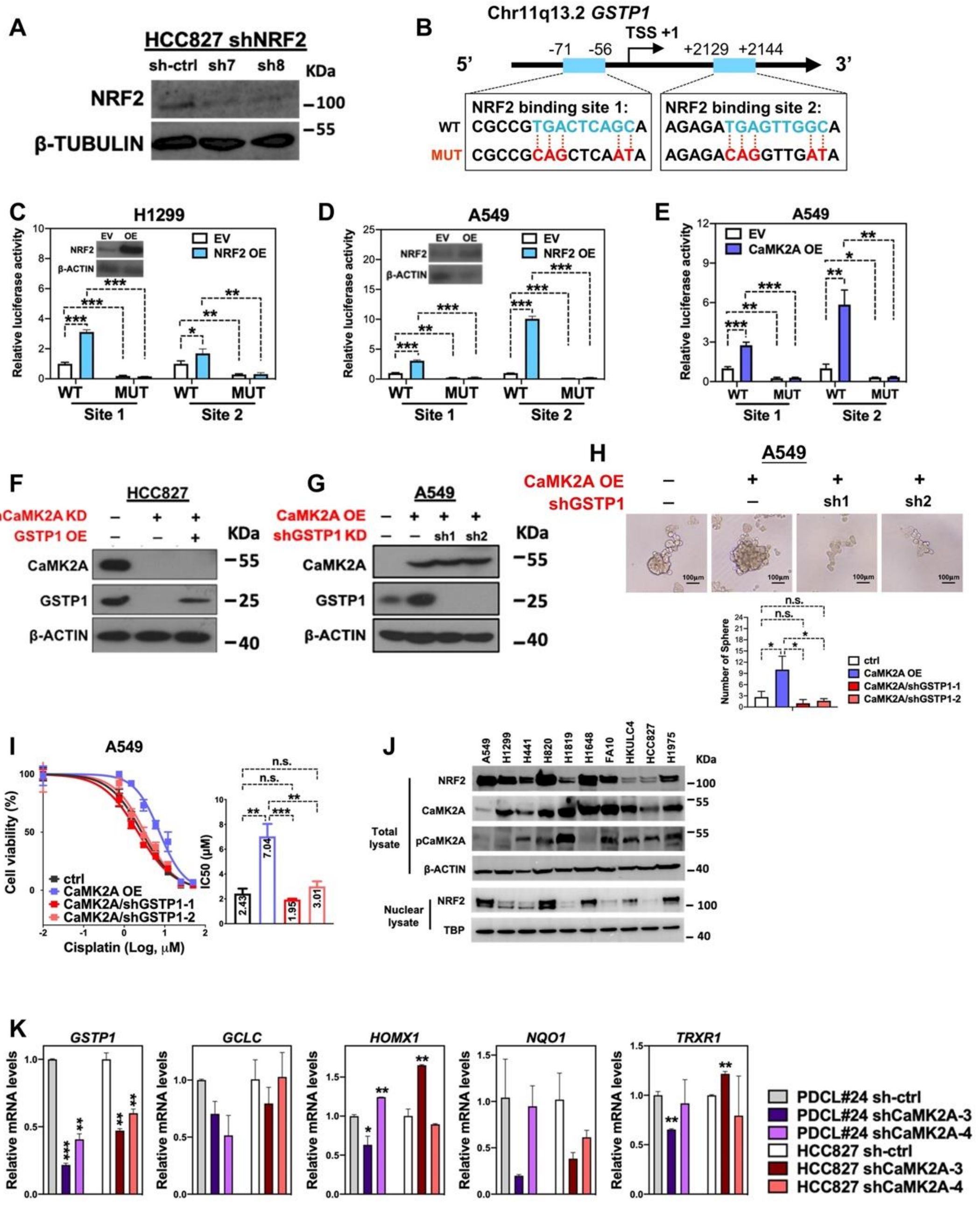

Supplementary figure 4. GSTP1 up-regulation was mediated by CaMK2A/NRF2 axis. (A) Western blot analysis of HCC827 cells with NRF2-KD. **(B)** NRF2 binding sites on *GSTP1* regulatory region and their corresponding mutant sequences. **(C-D)** Luciferase activity of *GSTP1* reporters with wild type or mutant NRF2-binding sites in H1299 cells **(C)** and A549 cells **(D)** with or without NRF2-OE, respectively. **(E)** Luciferase activity of *GSTP1* reporters with wild type or mutant NRF2-binding sites in A549 CaMK2A overexpressing cells. Western blot analysis of CaMK2A and GSTP1 expression in HCC827 cells **(F)**, and A549 cells **(G)**, with or without GSTP1 and/or CaMK2A manipulation. Effects of CaMK2A-OE and GSTP1-KD on sphere formation **(H)**, and cisplatin sensitivity by MTT assay **(I)**. **(J)** Western blot analysis of total NRF2, nuclear NRF2, CaMK2A and phospho-CaMK2A levels in LUAD cell line panel. **(K)** mRNA levels of NRF2 downstream

targets (GSTP*1, GCLC, HOMX1, NQO1, TXTR1*) in PDCL#24 and HCC827 cells with CaMK2A-KD, respectively. Data is presented as mean ± SD of triplicate measurements. * p<0.05, ** p<0.001, *** p<0.005 versus respective control by Student's t-test.

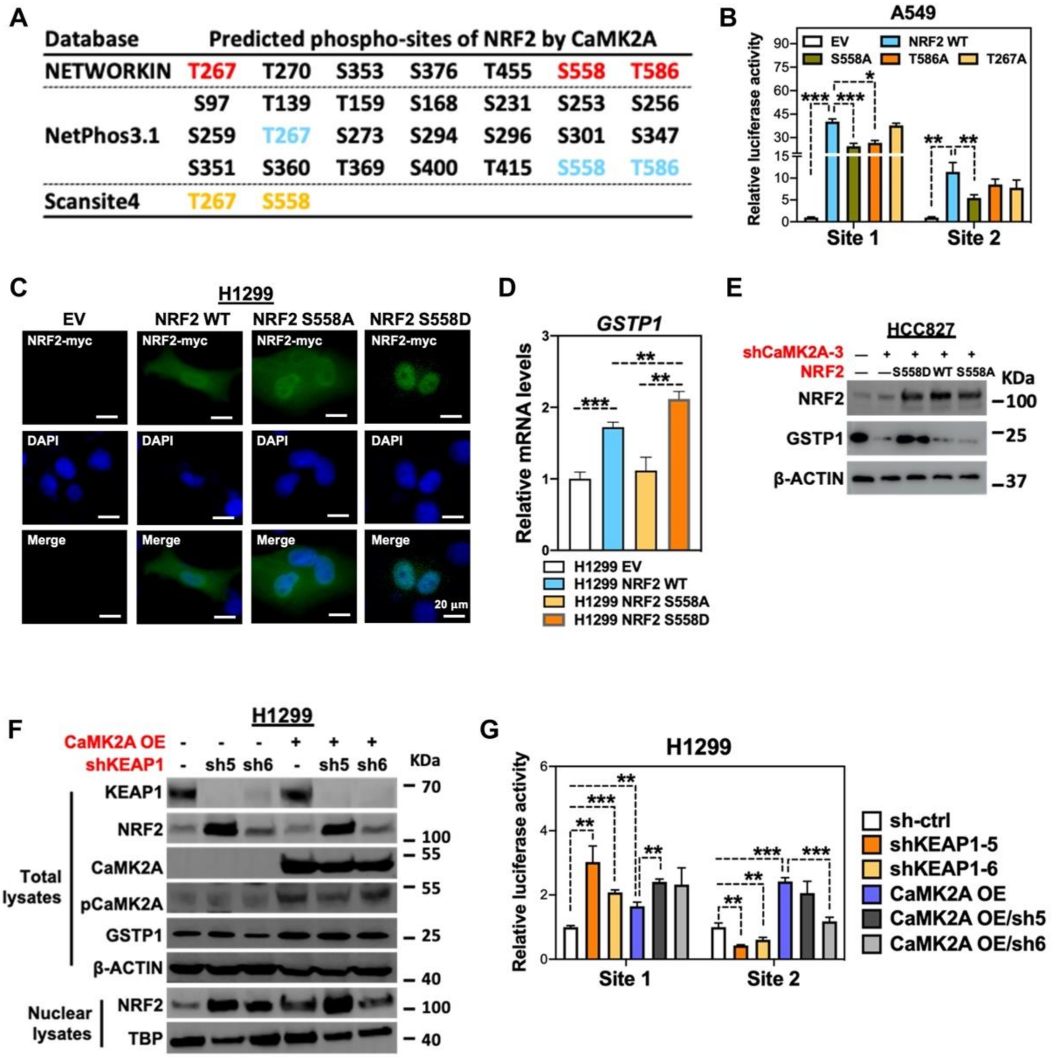

Supplementary figure 5. CaMK2A directly phosphorylated NRF2 at S558 residue. (A) Potential CaMK2A phosphorylation residues on NRF2 predicted by NETWORKIN, Netphos3.1 and Scansite4 online prediction tools. **(B)** Luciferase activity of *GSTP1* reporters in A549 cells with exogenous expression of wild type or mutant NRF2 (T267A, S558A, T586A). **(C)** Immunofluorescent staining of NRF2 localization in H1299 with empty vector (EV), NR2-WT, NRF2-S558A and NRF2-S558D overexpression, respectively. **(D)** GSTP1 expression in H1299 with empty vector, NR2-WT, NRF2-S558A and NRF2-S558D overexpression by qPCR. **(E)** Western blot analysis of GSTP1 expression in HCC827 CaMK2A-KD cells with NR2-WT, NRF2-S558A or NRF2-S558D overexpression. **(F)** Western blot analysis of NRF2, CaMK2A, phospho-CaMK2A T286, GSTP1 and nuclear NRF2 levels in H1299 cells with CaMK2A and/or KEAP1 manipulation. **(G)** Luciferase

activity of *GSTP1* reporters in H1299 cells with CaMK2A and/or KEAP1 manipulation. Data is presented as mean ± SD of triplicate measurements. * $p<0.05$, ** $p<0.001$, *** $p<0.005$ versus respective control by Student's *t*-test.

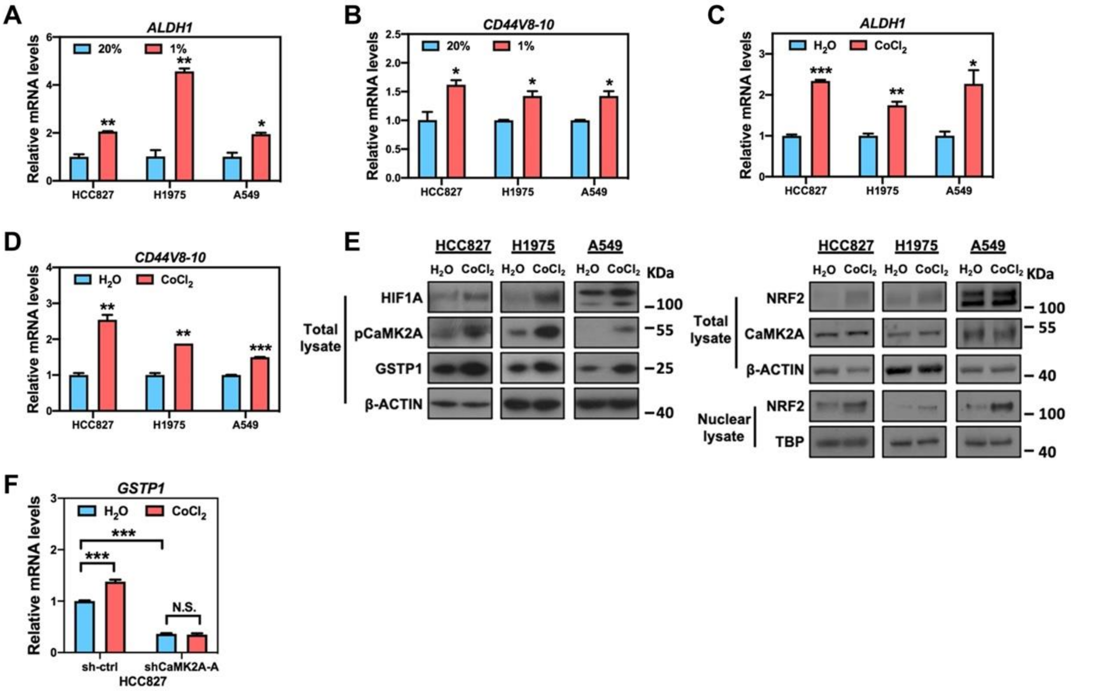

Supplementary figure 6. Hypoxia activated CaMK2A/NRF2/GSTP1 axis. Relative mRNA levels of *ALDH1* **(A)**, and *CD44V8-10* **(B)**, in LUAD cells incubated in 1% O_2 or 20% O_2. **(C-D)** Relative mRNA levels of *ALDH1* **(C)**, and *CD44V8-10* **(D)**, in LUAD cells treated with 100μ M $CoCl_2$. **(E)** Western blot analysis of HIF1A, NRF2, CaMK2A, phospho-CaMK2A T286, GSTP1 and nuclear NRF2 expressions in LUAD cell lines treated with or without 100 μM $CoCl_2$. **(F)** Relative mRNA levels of *GSTP1* in HCC827 cells with or without CaMK2A-KD under $CoCl_2$ treatment. Data is presented as mean ± SD of triplicate measurements. * $p<0.05$, ** $p<0.001$, *** $p<0.005$ versus respective control by Student's *t*-test.

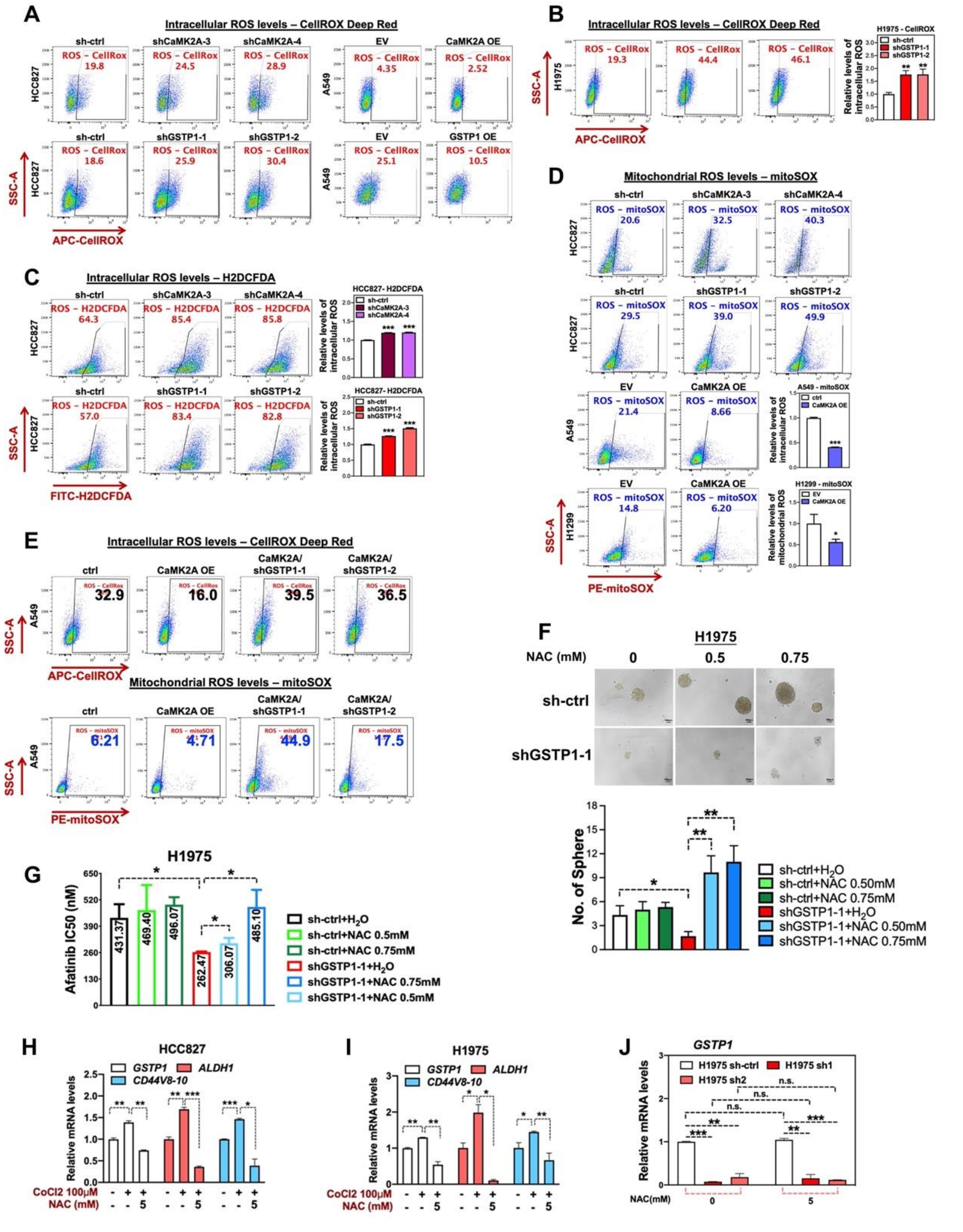

Supplementary figure 7. CaMK2A/GSTP1 axis regulated both intracellular and mitochondrial ROS levels. (A) Dot images represented intracellular ROS levels detected by CellRox Deep Red dye in HCC827 and A549 cells with either CaMK2A or GSTP1 manipulation. **(B)** Intracellular ROS levels detected by CellRox Deep Red dye in H1975 cells with GSTP1-KD. Histogram represented the relative ROS levels. **(C)** Intracellular ROS levels detected by H2DCFDA dye in HCC827 cells with either CaMK2A or GSTP1 manipulation. Histograms represented the relative ROS levels. **(D)** Mitochondrial ROS levels detected by mitoSOX dye in HCC827, H1975, A549, and H1299 cells with either CaMK2A or GSTP1

manipulation. Histograms represented the relative ROS levels. **(E)** Intracellular and mitochondrial ROS levels of A549 cells with CaMK2A and/or GSTP1 manipulation. **(F)** Tumorsphere formation assay of H1975 cells with or without GSTP1 silencing in the presence or absence of NAC. Representative bright field images of tumorspheres (upper panel) and histograms of sphere numbers (lower panel) were shown. **(G)** The effects of NAC co-treatment on drug sensitivity of H1975 cells with or without GSTP1-KD towards afatinib by MTT assay. **(H-I)** The mRNA levels of *GSTP1*, *ALDH1* and *CD44V8-10* in HCC827 **(H)** and H1975 **(I)** treated by 100μm of $CoCl_2$ or/and 5mM of NAC. **(J)** The effects of NAC treatment on *GSTP1* mRNA level of H1975 cells with or without GSTP1-KD. Data is presented as mean ± SD of triplicate measurements. * p<0.05, ** p<0.001, *** p<0.005 versus respective control by Student's *t*-test.

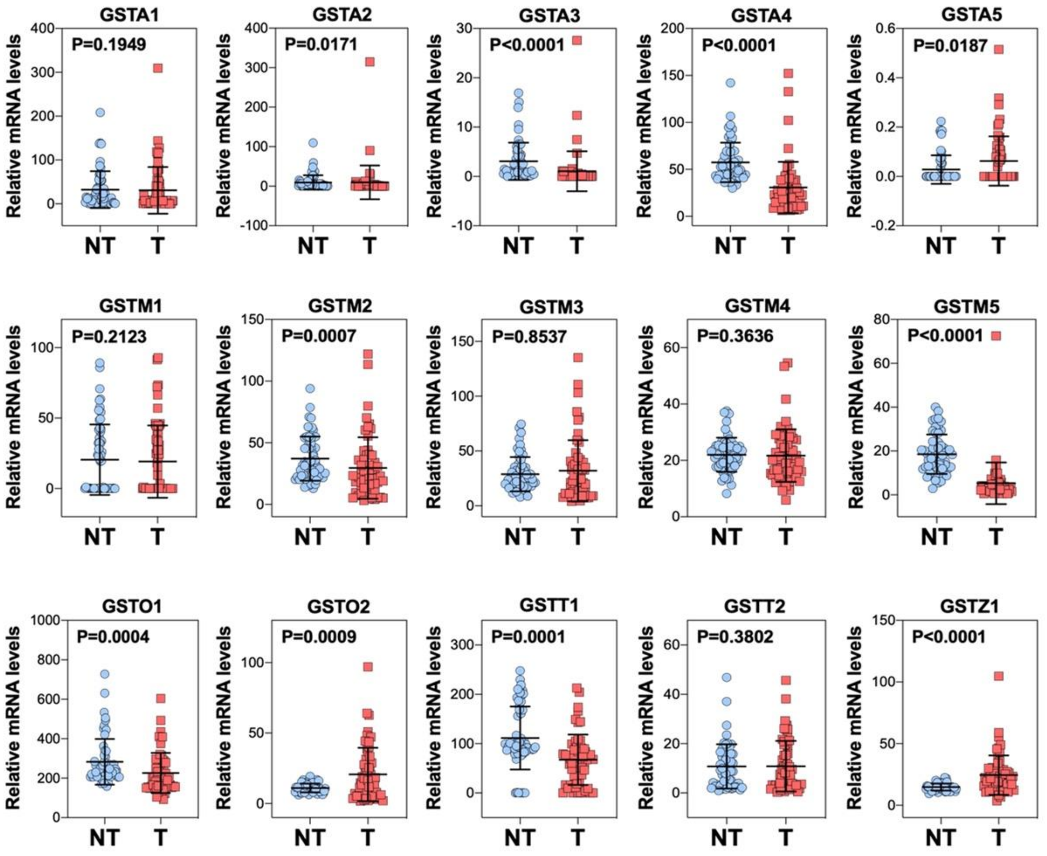

Supplementary Figure 8. Expression patterns of GST isoforms in lung cancer. Relative mRNA levels of GST isoforms in 58 paired lung cancer and non-tumor tissues according to TCGA lung cancer database.

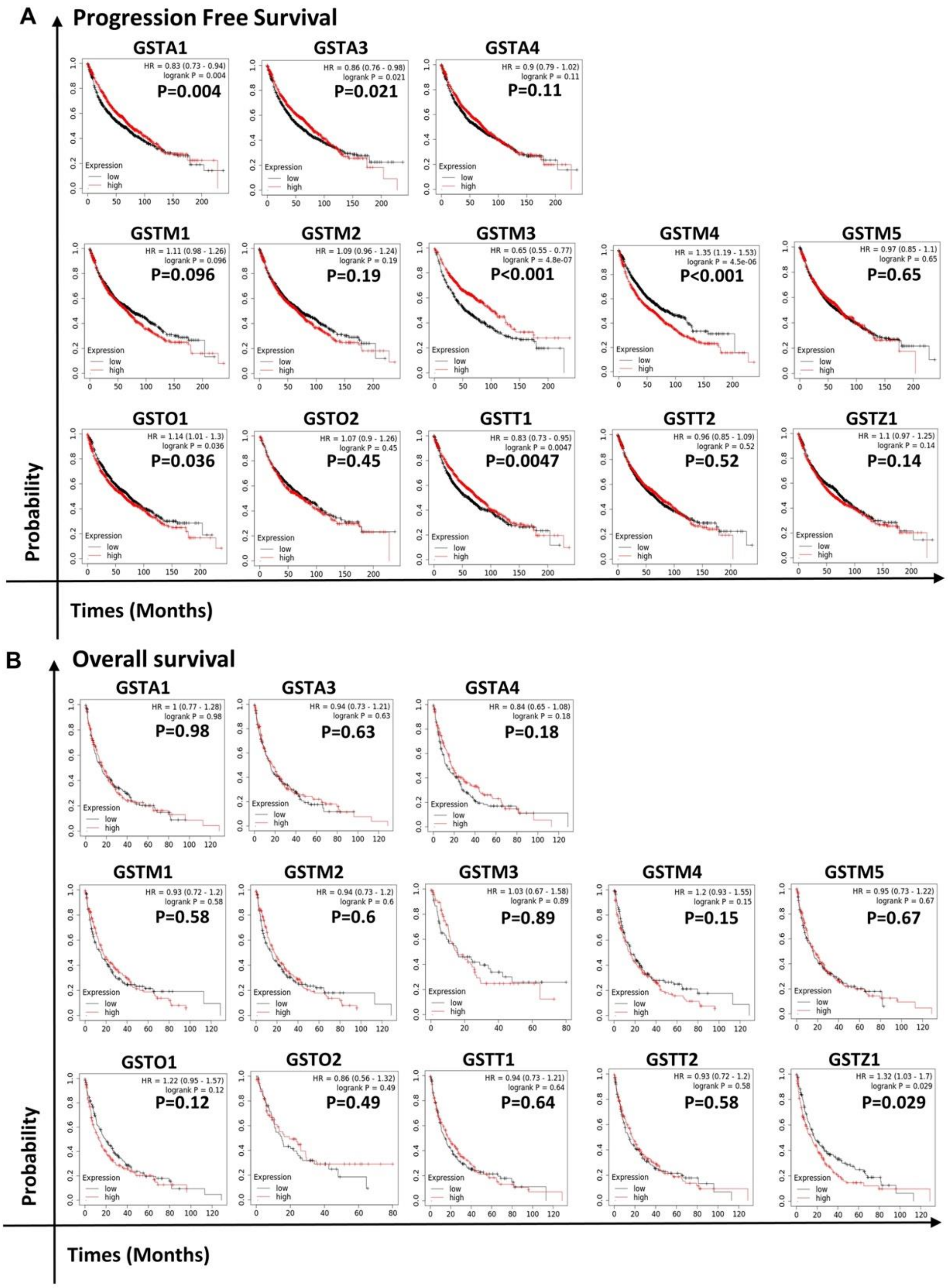

Supplementary figure 9. Clinical significance of GST isoforms in lung cancer. Data from the Kaplan-Meier Plotter database (www.kmplot.com/lung) were used to analyze the progression free **(A),** and overall **(B)** survivals with low/high GST levels stratified by the median expression.

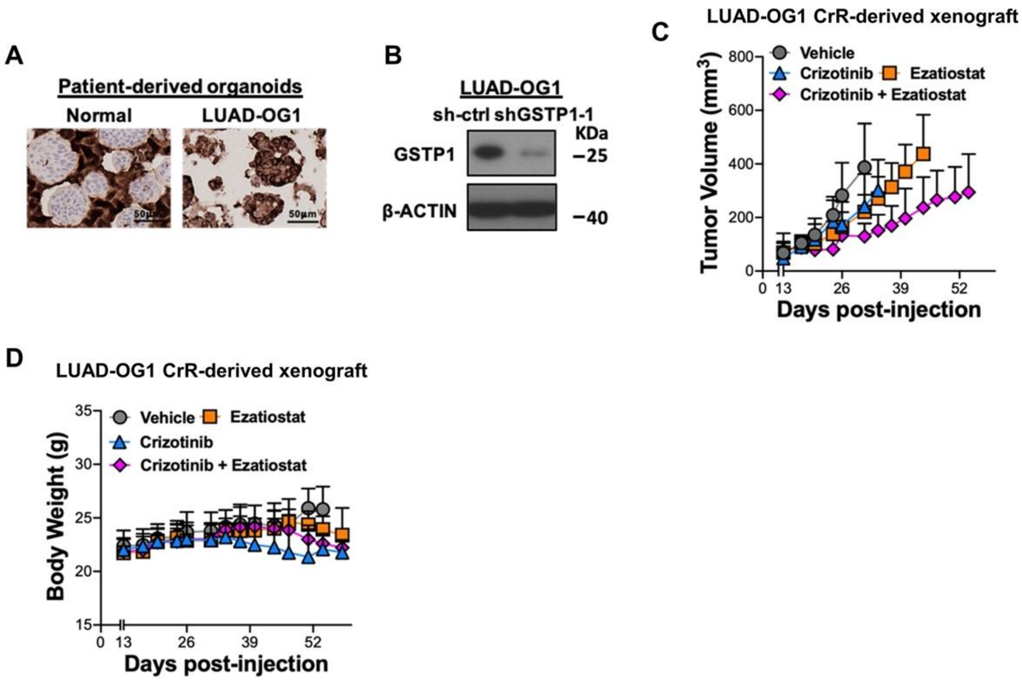

Supplementary figure 10. GSTP1 is potential therapeutic target against CSC in LUAD. (A) IHC staining of LUAD-OG1 and matched normal lung organoids illustrated positivity of ALK staining in LUAD-OG1. **(B)** Western blot analysis of GSTP1 expression in LUAD-OG1 with or without GSTP1-KD **(C)** Tumor growth curve during treatment. The end point of each group was taken as the number of post-injection days when the first mouse died in that group. **(D)** Graph of animal body weights during treatment. Data is presented as mean ± SD of triplicate measurements.

Supplementary Table 1

Primers and Oligos (5'-3')	
GSTP1 cDNA amplification	
F	TGCTCTAGAGCCACCATGCCGCCCTACACCGTG
R	CCGGAATTCTCACTGTTTCCCGTTGCCAT
QPCR primers_Gene name	
GSTP1	ACCTCCGCTGCAAATACATC
	GACAGCAGGGTCTCAAAAGG
ALDH1	ATGCTTCCGAGAGGGGGCGA
	CCCAACCTGGACAGTAGCGCA
CD44	TCCAACACCTCCCAGTATGACA
	GGCAGGTCTGTGACTGATGTACA
CD44V3	ACGTCTTCAAATACCATCTC
	CCAAGATGATCAGCCATTCTGG
CD44V4	ACCACACCACGGGCTTTTGACC

	GGGTTCCACTGGGTCCAGTCCT
CD44V6	GAAGAAACAGCTACCCAGAAGGAACAG
	GCCAAGAGGGATGCCAAGATG
CD44V8	TCAGCCTACTGCAAATCCAA
	GAGGTCCTGTCCTGTCCAAA
CD44V8-10	AGAATCCCTGCTACCAATATGGACTC
	AGGTCACTGGGATGAAGGTC
CD44V10	GCAGCACTTCAGGAGGTTACAT
	ATGATTTGGGTCTCTTCTTCCA
B2M	AGGCTATCCAGCGTACTCCA
	GGCATCTTCAAACCTCCAT
RPL13A	GCCCTACGACAAGAAAAAGGG
	TACTTCCAGCCAACCTCGTGA
Mutagenesis primers	
NRF2 T267A F<br>NRF2 T267A R	TGAATTTAATGAGTTCACTGCCAACTGGTTGGGGTCTTCT<br>AGAAGACCCCAACCAGTTGGCAGTGAACTCATTAAATTCA
NRF2 S558A F<br>NRF2 S558A R	CTGAAAACTTCGAGATATAAGGTGGCGAGTTGTTTTTCAGTAG GTGAAG<br>CTTCACCTACTGAAAAACAACTCGCCACCTTATATCTCGAAGT TTTCAG
NRF2 T586A F<br>NRF2 T586A R	AAACATTGCCATCTCTTGCTTGCTGCAGGGAGTATTC<br>GAATACTCCCTGCAGCAAGCAAGAGATGGCAATGTTT
